# A novel transcriptional regulator of L-arabinose utilization in human gut bacteria

**DOI:** 10.1093/nar/gkv1005

**Published:** 2015-10-04

**Authors:** Changsoo Chang, Christine Tesar, Xiaoqing Li, Youngchang Kim, Dmitry A. Rodionov, Andrzej Joachimiak

**Affiliations:** 1 Midwest Center for Structural Genomics, Argonne National Laboratory, Argonne, IL 60439, USA; 2 Structural Biology Center, Biosciences Division, Argonne National Laboratory, Argonne, IL 60439, USA; 3 Sanford-Burnham Medical Research Institute, La Jolla, CA 92037, USA; 4 A. A. Kharkevich Institute for Information Transmission Problems, Russian Academy of Sciences, Moscow 127994, Russia; 5 Department of Biochemistry and Molecular Biology, University of Chicago, Chicago, IL 60637, USA

## Abstract

Carbohydrate metabolism plays a crucial role in the ecophysiology of human gut microbiota. Mechanisms of transcriptional regulation of sugar catabolism in commensal and prevalent human gut bacteria such as *Bacteroides thetaiotaomicron* remain mostly unknown. By a combination of bioinformatics and experimental approaches, we have identified an NrtR family transcription factor (BT0354 in *B. thetaiotaomicron*, BtAraR) as a novel regulator controlling the arabinose utilization genes. L-arabinose was confirmed to be a negative effector of BtAraR. We have solved the crystal structures of the apo and L-arabinose-bound BtAraR proteins, as well as the complex of apo-protein with a specific DNA operator. BtAraR forms a homodimer with each subunit comprised of the ligand-binding Nudix hydrolase-like domain and the DNA-binding winged-helix-turn-helix (wHTH) domain. We have identified the residues involved in binding of L-arabinose and recognition of DNA. The majority of these residues are well conserved in the AraR orthologs in *Bacteroidetes*. In the structure of the BtAraR–DNA complex, we found the unique interaction of arginine intercalating its guanidinum moiety into the base pair stacking of B-DNA. L-arabinose binding induces movement of wHTH domains, resulting in a conformation unsuitable for DNA binding. Our analysis facilitates reconstruction of the metabolic and regulatory networks involved in carbohydrate utilization in human gut *Bacteroides*.

## INTRODUCTION

The human gastrointestinal tract is predominantly a bacterial ecosystem that is largely represented by micro-organisms from two divisions—the *Bacteroidetes* and the *Firmicutes* (1,2). The *Bacteroides* genus includes commensal Gram-negative anaerobic bacteria that play a fundamental role in the breakdown of dietary polysaccharides in the host intestine (3,4), and that have evolved a divergent array of genes involved in sensing, regulation and polysaccharide degradation and utilization (5). Decomposition and further utilization of complex and diverse oligosaccharides play a critical role in the ecophysiology of human gut microbiota. The ability to confidently reconstruct respective biochemical and regulatory networks from genomic and metagenomic data would strongly impact predictive modeling of microbial communities and their interactions with the host in health and disease. However, presently this ability is hampered by a limited knowledge of functions of their key components (transporters, regulators, enzymes). Combining structural genomics with predictive bioinformatics and experimental functional characterization allows us to fill in major gaps in this knowledge and enables accurate reconstruction of carbohydrate metabolism in previously uncharacterized microbial species and communities.

Many bacteria use L-arabinose as a source of carbon and energy. The L-arabinose utilization pathway and its transcriptional regulation have been studied extensively in several model microorganisms. The three consecutive steps of the L-arabinose catabolic pathway are catalyzed by L-arabinose isomerase AraA, L-ribulokinase AraB and L-ribulose-phosphate epimerase AraD. In *Escherichia coli*, growth on L-arabinose involves expression of three unlinked L-arabinose-inducible operons; one encodes enzymes (*araBAD*) and the other two encode proteins for arabinose uptake (*araFGH* and *araE*). In the presence of L-arabinose, the transcriptional factor AraC activates the *ara* promoters in *E. coli* (6). In *Bacillus subtilis* and other Gram-positive bacteria from the *Firmicutes* phylum, the utilization of L-arabinose is controlled by the transcription factor AraR (7). In the absence of the effector L-arabinose, apo-AraR binds to operator sites in the promoter regions of the *ara* genes and serves as a repressor of their transcription. The *B. subtilis* AraR repressor consists of a GntR-type DNA-binding domain in the N-terminal region and a C-terminal effector-binding domain homologous to the LacI family of regulators (8). The structural studies for AraC from *E. coli* (9–12) and AraR from *B. subtilus* (8,13) confirmed that these two L-arabinose-responsive transcription factors belong to different families, AraC and GntR/LacI (14), respectively. In this study we identified and characterized a novel L-arabinose-responsive transcription factor found in the *Bacteroides* species, which belongs to the NrtR family of Nudix-related transcriptional regulators (15).

The NrtR family transcription factors are characterized by an N-terminal Nudix hydrolase-like effector-binding domain and a C-terminal DNA-binding domain. Several NrtR regulators from diverse phylogenetic groups of bacteria were previously characterized as repressors of genes implicated in the NAD cofactor metabolism (15). ADP-ribose, the product of glycohydrolytic cleavage of NAD, suppresses the DNA binding activity of NrtR proteins from *Shewanella oneidensis* and *Synechocystis* spp. (15), whereas the NrtR family regulator NdnR in *Corynebacterium glutamicum* responds to NAD (16). The structure of NrtR protein from *S. oneidensis* (SoNrtR) has been solved both in the apo-form and in complex with either ADP-ribose or with a 27-bp DNA fragment containing the NrtR recognition sequence (17). However, the ADP-ribose-bound SoNrtR structure contains only the N-terminal ligand-binding domain. The NrtR family of regulators is characterized by highly variable DNA-binding sequence motifs present in different groups of bacteria (15). This observation correlates with the absence of sequence conservation of the DNA-interacting residues observed in the DNA-binding domain.

*Bacteroides thetaiotaomicron* of the *Bacteroides* genus is one of the most abundant and intensively studied commensal species that colonize the mammalian gastrointestinal tract and form extensive symbiotic relationships with the host (18,19). The bioinformatics analysis of a divergent branch of the NrtR family represented by two previously uncharacterized proteins in *B. thetaiotaomicron*, BT0354 (BtAraR) and BT0791 (BtXylR), suggests that these regulators possibly control the catabolic pathways for two pentose sugars, L-arabinose and D-xylose (20). In this work, genome-scale regulon inference using the comparative genomics approach revealed that the NrtR family AraR regulators in *Bacteroides* and *Prevotella* spp. control not only the L-arabinose catabolic operons, but also several other gene loci involved in the utilization of arabinose-containing polysaccharides. The predicted function of BtAraR was validated by *in vitro* binding assays. Further structural characterization of this novel arabinose-responsive regulator provides new insights into sugar-mediated mechanisms of BtAraR transcription regulation. The comparison of the DNA- and L-arabinose-bound forms shows how L-arabinose binding reduces BtAraR affinity for its specific DNA operator sequence.

## MATERIALS AND METHODS

### Genomic reconstruction of regulons

We applied the integrative comparative genomics approach to reconstruct the AraR regulons in *Bacteroides* species (as implemented in the RegPredict Web server, http://regpredict.lbl.gov) (21). The approach combines identification of candidate regulator binding sites with cross-genomic comparison of regulons and with functional context analysis of candidate target genes. The upstream regions of arabinose utilization genes in 17 *Bacteroides* genomes (representing a non-redundant set of species excluding closely related strains) were analyzed using a DNA motif recognition program (the ‘Discover Profile’ procedure implemented in RegPredict) to identify the conserved AraR-binding DNA motif. After construction of a positional-weight matrix for the AraR motif, we searched for additional AraR-binding sites in the analyzed *Bacteroides* genomes and performed a consistency check or cross-species comparison of the predicted AraR regulons. Scores of candidate sites were calculated as the sum of positional nucleotide weights. The score threshold was defined as the lowest score observed in the training set. Sequence logo for the derived DNA-binding motif was drawn using the WebLogo package (22). The details of the reconstructed AraR regulon are captured and displayed in RegPrecise, a specialized database of bacterial regulons (http://regprecise.lbl.gov) (23), as a part of the *Bacteroides* collection. Information on co-regulation of the AraR-controlled operons by other transcription factors, including various carbohydrate-specific hybrid two-component systems (HTCSs), was extracted from the RegPrecise database.

### Protein cloning, expression and purification

The BT0354 gene was amplified from *B. thetaiotaomicron* genomic DNA with *KOD* DNA polymerase using conditions and reagents provided by the vendor (Novagen, Madison, WI, USA). The gene was cloned into a pMCSG68 vector by using a modified ligation-independent cloning protocol (24,25). The pMCSG68 vector bearing a TEV protease cleavage site creates a construct with a cleavable His_6_-tag fused onto the N-terminus of the target protein and adds three artificial residues (Ser-Asn-Ala) on that end. The gene was overexpressed in *E. coli* BL21 (DE3) carrying plasmids pMAGIC that encode one rare *E. coli* tRNA (Arg [AGG/AGA]) and pRK1037 (Scientific Reagents, Inc.).

The cells were grown using selenomethionine (SeMet) containing enriched M9 medium and under conditions known to inhibit methionine biosynthesis. The cells were grown at 37°C to an OD_600_ of ∼0.6 and protein expression was induced with 0.5 mM IPTG. The cells were grown overnight with shaking at 18°C. The harvested cells were resuspended in five volumes of lysis buffer (50 mM HEPES pH 8.0, 500 mM NaCl, 20 mM imidazole, 10 mM β-mercaptoethanol and 5% v/v glycerol) and stored at −20°C. The thawed cells were lysed by sonication after the addition of inhibitors of proteases (Sigma, P8849) and 1 mg/ml lysozyme. The lysate was clarified by centrifugation at 30 000 *g* (RC5C-Plus centrifuge, Sorval) for 60 min, followed by filtration through 0.45 and 0.22 μm in-line filters (Gelman). Immobilized metal affinity chromatography (IMAC-I) using a 5-ml HiTrap Chelating HP column charged with Ni^+2^ ions followed by buffer-exchange chromatography on a HiPrep 26/10 desalting column (both GE Healthcare Life Sciences) were performed using an ÄKTAxpress system (GE Healthcare Life Sciences). His_6_-tag was cleaved using the recombinant TEV protease expressed from the vector pRK508. The protease was added to the target protein in a ratio of 1:30 and the mixture was incubated at 4°C for 48 h. The BtAraR protein was then purified using a 5 ml HiTrap Chelating column charged with Ni^+2^ ions. The protein was dialyzed in 20 mM HEPES pH 8.0, 250 mM NaCl, 2 mM DTT and concentrated using a Centricon Plus-20 Centrifugal Concentrator (Millipore) to 54 mg/ml.

### DNA binding assays

The interaction of the purified recombinant BtAraR protein with its specific DNA-binding sites in *B. thetaiotaomicron* was assessed using an electrophoretic mobility-shift assay (EMSA). Complementary DNA oligos containing the predicted BtAraR binding sites from the BT0356 and BT0365 gene promoter regions were synthesized by Integrated DNA Technologies. The 65-bp DNA oligo with a fragment of the promoter region of the BT0356 (*araM*) gene, cccccATATAAGAGTGTATTTGATACACCAAACAAAAGTGTTACTTTTACACCCAAAATAccccc, contained two predicted AraR-binding sites (underlined) and was flanked on each side by five cytosine residues (lower case). The 41-bp DNA oligo with a fragment of the promoter region of the BT0365 gene, cccccTACTCAAAGTGTAAAAAAGACACTTATATAAccccc, contained a single predicted AraR-binding site (underlined). One strand of the oligo was 5′-labeled by biotin, whereas the complimentary strand was unlabeled. The double-stranded labeled DNA fragments were obtained by annealing the labeled oligonucleotides with unlabeled complementary oligonucleotides at a 1:10 ratio. The labeled DNA fragments (0.2 nM) were incubated with increasing concentrations of the purified BtAraR protein (0.25–1 μM) in a total volume of 20 µl of the binding buffer containing 20 mM Tris–HCl (pH 8.0), 150 mM KCl, 5 mM MgCl_2_, 1 mM DTT, 0.05% NP-40 and 2.5% glycerol. After 30 min of incubation at 37°C, the reaction mixtures were separated by electrophoresis on a 1.5% agarose gel in 0.5× TB (60 min, 90 V). The DNA was transferred by the capillary method onto a Hybond-N^+^ membrane and fixed by UV cross-linking. Biotin-labeled DNA was detected with the LightShift chemiluminiscent EMSA kit (Thermo Fisher Scientific Inc, Rockford, IL, USA). To identify effectors of AraR, additional EMSA experiments were performed to test the effect of carbohydrates on the DNA-binding affinity of the regulator. The BtAraR protein (1 μM) and the BT0356 DNA fragment (0.2 nM) were incubated with increasing concentrations of L-arabinose and D-xylose in the incubation mixture (0.01–2 mM). A DNA fragment containing the shuffled sequence of the BT0356 DNA fragment flanked by five-cytosine regions on both sides was used as a negative control, cccccACTATAATTAACCAATTAATCTAATCCAACGGTTACATAGGAGGTACTAATATA Accccc.

### Protein crystallization and data collection

Initial crystallization screens were set up using the Mosquito robot (TTP Labtech) and the sitting drop vapor diffusion technique in a 96-well CrystalQuick plate (Greiner). The apo-BtAraR crystallized in two crystal forms. The apo-1-BtAraR protein was crystallized by vapor diffusion in hanging drops by mixing 1 μl of the protein solution with 1 μl of reservoir solution (0.4 M NaH_2_PO_4_, 1.6 M Na_2_HPO_4_, 0.2 M NaCl, 0.1M Tris-Cl, pH 8.2) and equilibrated at 289 K over 500 μl of this solution. Crystals were flash-cooled in liquid nitrogen with reservoir solution plus 25% glycerol as a cryoprotectant prior to data collection. The apo-2-BtAraR protein was crystallized in MCSG3 suite condition F4 (2.1 M DL-malic acid pH 7.0). For the AraR–DNA complex crystal, a 27-bp DNA oligonucleotide (GCAAAAGTGTTACTTTTACACCCATGC) was used that corresponds to the BT0356 (*araM*) gene 22-nt promoter region (underlined). Two synthetic complementary oligonucleotides were annealed and used in crystallizations. The crystallization condition was screened by 96-well format, after which the protein to DNA ratio was optimized using the hanging drop format with the same well solution but with different ratios. The final optimized molar ratio for protein to DNA is 1:2. The BtAraR–DNA complex was crystallized in Natrix suite condition G2 (20 mM magnesium chloride hexahydrate, 50 mM MOPS pH7.0, 55% (v/v) tacsimate pH 7.0, 5mM hexammine cobalt(III) chloride). The BtAraR–L-arabinose complex was crystallized in MCSG1 suite condition C3 (0.2 M magnesium formate pH 5.9, 20% (w/v) PEG 3350).

Diffraction data were collected at 100K on ADSC quantum Q315r charged coupled device detector in the 19-ID beamline of the Structural Biology Center at the Advanced Photon Source, Argonne National Laboratory (26). Single wavelength anomalous dispersion (SAD) data near the selenium absorption peak was collected from a SeMet-substituted protein. The diffraction data were processed by using the HKL3000 suite of programs (27). Data collection statistics are summarized in Table 1.

**Table 1.** Data collection and refinement statistics

	Apo 1	Apo 2	DNA complex	Ara-bound
Wavelength (Å)	0.9794	0.9793	0.9790	0.9792
Spacegroup	R3	I2_1_3	P23	P2_1_
Cell parameters (Å, °)	*a* = *b* = 176.4 *c* = 118.4	*a* = *b* = *c* = 123.7	*a* = *b* = *c* = 163.0	*a* = 59.1 *b* = 49.4 *c* = 90.2 *β* = 107.1
Resolution range (Å)	50–2.35 (2.39–2.35)^a^	50–2.56 (2.58–2.56)^a^	50–3.05 (3.08–3.05)^a^	50–1.95 (1.98–1.95)^a^
Unique reflections	52,069	10,038	27,060	33,755
Multiplicity	3.7 (3.4)^a^	10.8 (11.0)^a^	5.2 (5.2)^a^	3.5 (2.9)^a^
Completeness (%)	91.4 (43.3)^a^	97.5 (78.4)^a^	97.9 (86.9)^a^	92.0 (45.9)^a^
Mean I/sigma(I)	10.9 (3. 7)^a^	18.9 (4.5)^a^	12.1 (3.1)^a^	20.0 (5.6)^a^
R_merge_	0.117 (0.831)^a^	0.114 (0.992)^a^	0.110 (0.851)^a^	0.041 (0.345)^a^
R_pim_	0.084 (0.511)^a^	0.036 (0.311)^a^	0.051(0.484)^a^	0.039 (0.243)^a^
CC*^a^	0.861	0.940	0.889	0.967
CC1/2^a^	0.588	0.792	0.653	0.877
R -work	0.195 (0.250)^a^	0.183 (0.258)^a^	0.200 (0.308)^a^	0.164 (0.190)^a^
R-free	0.233 (0.298)^a^	0.222 (0.323)^a^	0.230 (0.362)^a^	0.220 (0.247)^a^
Number of atoms	7488	1837	4762	3902
macromolecules	7213	1802	4726	3600
ligands	16	8	20	31
water	259	27	16	271
r.m.s.d.				
bond length	0.010	0.009	0.010	0.009
angles	1.25	1.34	1.40	1.33
Ramachandran				
favored (%)	99	98	96	99
outliers (%)	0	0.45	0.22	0.23
Molprobity clashscore	1.67	1.41	5.75	1.56
Average B-factor	39.2	35.6	65.4	33.5
macromolecules	39.5	35.8	65.5	33.4
ligand	28.6	37.2	67.6	36.4
solvent	31.3	24.7	47.7	34.5

^a^Values in the highest resolution shell.

### Structure determination, refinement and analysis

All structures were solved by the SAD method using selenium near absorption peak data. All procedures for SAD phasing, phase improvement by density modification, and initial protein model building were done by structure module of the HKL3000 software package (27). The mean figures of merit of the phase sets were as follows: for apo-1-BtAraR it was 0.282 for 50–2.70 Å data, for apo-2-BtAraR it was 0.317 for 50–2.92 Å data, for the BtAraR–DNA complex it was 0.315 for 50–3.11 Å data and for the L-arabinose–BtAraR complex it was 0.278 for 50–1.95 Å data. These statistics were improved after density modification (DM (28)) to FOM of 0.738, 0.801, 0.865 and 0.807 for apo-1–BtAraR, apo-2–BtAraR, BtAraR–DNA complex, and L-arabinose-bound form, respectively, for corresponding resolution ranges. For the apo-1–BtAraR model, the Arp/wArp (29) built 645 out of 900 residues and 377 of them were docked with their sequence. For the apo-2–BtAraR model, buccaneer (30) was used and found 221 out of 225 residues and fitted 179 residues in the sequence. For the BtAraR–DNA complex model, 465 residues (57 residues with side chains) were built using resolve (31) in model building module, but DNA is also traced as protein, so a full complex model was built using COOT (32). Arp/wArp was used for model building of the L-arabinose–BtAraR complex and found 359 out of 450 residues (348 residues with side chains). All models were rebuilt with the graphics program COOT, and between each cycle of rebuilding, the models were refined by REFMAC5 from the CCP4 suite (33,34) or PHENIX (35). The geometrical properties of the model were assessed by COOT and Molprobity (36). DNA geometry was calculated using Curves+ (37) and DNA protein interaction was calculated using NUCPLOT (38).

## RESULTS AND DISCUSSION

### Genomic reconstruction and prediction of functional specificity in a novel branch of the NrtR family

**Figure 1.**
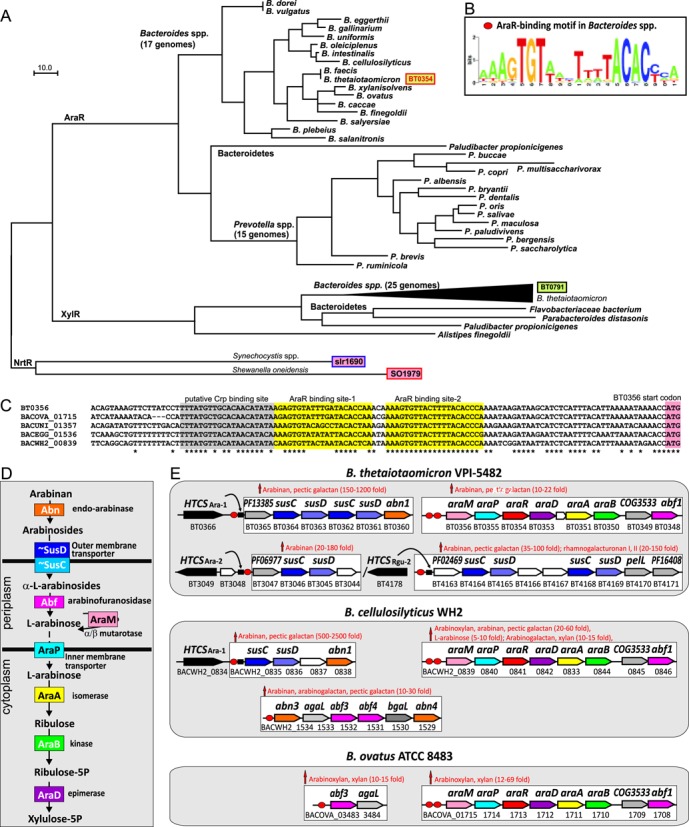
Comparative genomics reconstruction of AraR regulons in *Bacteroides* spp. (**A**) Maximum likelihood phylogenetic tree of NrtR family regulators from the *Bacteroidetes* phylum. The AraR and XylR regulators from *Bacteroides thetaiotaomicron* (33% identity to each other, shown in green boxes) belong to two different branches of the NrtR family regulators in the *Bacteroidetes*. Two previously characterized ADP-ribose-responsive NrtR regulators from *Shewanella oneidensis* and *Synechocystis* spp. are shown in pink boxes. Proteins with solved crystal structures are shown in red frames. (**B**) Sequence logo for AraR-binding motif in *Bacteroides* spp. The logo was constructed using ∼50 candidate AraR sites from 17 *Bacteroides* spp. (**C**) Multiple sequence alignment of promoter regions of BT0356 (*araM*) and its orthologs in *Bacteroides* spp. Tandem AraR-binding sites and a putative binding site of a Crp-like regulator are highlighted. (**D**) AraR-regulated metabolic pathway for utilization of L-arabinose and its polymers. The pathway includes extracytoplasmic hydrolytic enzymes (Abn, Abf), transporters through the outer membrane (SusCD) and the inner membrane (AraP), periplasmic L-arabinose mutarotase (AraM) and cytoplasmic L-arabinose catabolic enzymes (AraA, AraB, AraD). (**E**) Reconstructed AraR regulons in three *Bacteroides* genomes. Candidate AraR-binding sites are shown by red circles; sequences of AraR sites in these and other genomes are listed in Supplementary Table S1. Genes/potential operons are shown by arrows grouped in large white boxes. Genes with the same functional roles are marked in matching colors. Hypothetical genes are shown by gray and white arrows. Hybrid two-component system (HTCS) regulators and their candidate binding sites are shown by black arrows and squares, respectively. Transcriptional induction of AraR-controlled operons by L-arabinose and arabinose-containing polymers is summarized using the previous transcriptomics studies (41,42).

Orthologs of the *B. thetaiotaomicron* AraR (BT0354) and XylR (BT0791) regulators were identified only within the *Bacteroidetes* phylum. AraR is present in 17 *Bacteroides* and 15 *Prevotella* spp., whereas XylR was found in 25 *Bacteroides* spp. and in several other *Bacteroidetes* (Figure 1A). We noted a strong tendency of *araR* and *xylR* genes to cluster on the chromosome with the arabinose and xylose utilization genes, respectively. Among 30 non-redundant *Bacteroides* species analyzed in this work, the AraR regulators and arabinose catabolic pathway genes were found in 17 species, while the other 13 *Bacteroides* species potentially have lost the arabinose catabolic genes. Multiple alignments of these AraR proteins revealed high overall conservation of their primary sequences (Supplementary Figure S1), suggesting the AraR orthologs are functionally identical with potentially preserved specificities toward the effector molecule and DNA sites. The Nudix signature motif GX_5_-EX_7_REUXEEXGU (where U is a hydrophobic residue and X is any residue) is strictly conserved in all known active Nudix hydrolases, but it is impaired in the NrtR regulators of NAD metabolism, several of which are known to be enzymatically inactive (15). Similarly to NrtR regulators, the Nudix signature motif is not conserved in the AraR proteins from *Bacteroidetes*, where two or three glutamate residues are substituted with other amino acids (Supplementary Figure S1). These observations suggest that AraR regulators are also enzymatically inactive and utilize their Nudix hydrolase-like domains for ligand binding.

To identify DNA-binding sites and reconstruct AraR regulons in *Bacteroides* spp., we applied the comparative genomics approach that combines identification of candidate binding sites with cross-genomic comparison of regulons (39,40). We applied a motif recognition program to the upstream promoter regions of the arabinose utilization operons from *Bacteroides* spp., resulting in identification of a conserved 21-bp DNA motif, which constitutes the candidate AraR binding site (Figure 1B). The *araMPRDAB* operons in *B. thetaiotaomicron* and 14 other *Bacteroides* species are preceded by two tandem semipalindromic AraR sites separated by either 3 bp (as in the majority of genomes, see Figure 1C) or by 42 nt (as in *B. vulgatus* and *B. dorei*). As a result, the reconstructed AraR regulons in all *araR*-containing genomes of *Bacteroides* and *Prevotella* include the *araMPRDAB* operons (Supplementary Table S1). In all *Prevotella* and most *Bacteroides* species, these AraR-controlled operons are expanded by an additional gene at the distal end of the operon, *abf3*, encoding α-L-arabinofuranosidase, which specifically hydrolyzes non-reducing residues from arabinose-containing oligosaccharides (Figure 1D).

Genomic searches for similar DNA sites resulted in identification of additional operons that can potentially be co-regulated by AraR in the *Bacteroides* genomes (Figure 1E). These additional AraR-regulated operons encode multiple paralogs of α-L-arabinofuranosidases (Abf from the GH51 family), arabinan endo-arabinosidases (Abn from the GH43 family) and other glycosyl hydrolases from the GH2 and GH97 families (AgaL, BgaL), as well as several TonB-dependent outer membrane transport systems (SusC-SusD) (Supplementary Table S1). Many of the above listed functions encoded by individual polysaccharide utilization gene loci (PULs) are also controlled by their cognate transcriptional regulators from the HTCS family. Thus we found that several previously described HTCS regulons (20) overlap with the reconstructed AraR regulons in *Bacteroides* spp. (Supplementary Table S1). Three PULs in *B. thetaiotaomicron* are under dual control of both HTCS and AraR regulators (Figure 1E). The predicted AraR-regulated operons are involved in the uptake and catabolism of L-arabinose and the decomposition of arabinose-containing polysaccharides (e.g., arabinan, arabinoxylan, pectic galactan) to oligosaccharides, their translocation into the periplasm, and further hydrolysis (Figure 1D).

The results of previous transcriptional profiling for *B. thetaiotaomicron* and *B. ovatus* in response to plant and host glycans (41) and for *B. cellulosilyticus* grown in the presence of 31 distinct mono- and polysaccharides (42) were compared with the reconstructed AraR regulons. First, all predicted AraR-regulated operons in the above three *Bacteroides* species were significantly upregulated when grown on one or several arabinose-containing polysaccharides such as arabinan, arabinogalactan, pectic galactan, arabinoxylan and xylan (Figure 1E). Second, genes from the arabinose utilization operon in *B. cellulosilyticus* were 5–10-fold upregulated when grown on L-arabinose as compared to D-glucose, thus confirming the AraR regulon is indeed induced by L-arabinose *in vivo*.

We hypothesize that these novel AraR regulators from the NrtR family respond to one or several intermediates of the L-arabinose utilization pathway. We further tested these predictions by focused DNA binding assays and solving crystal structures of BtAraR in complexes with specific DNA or L-arabinose. The obtained structure-function information was then generalized to elucidate evolution and structural basis of sugar specificity in the novel branch of regulators from the NrtR family.

### Experimental validation of the predicted BtAraR regulon

**Figure 2.**
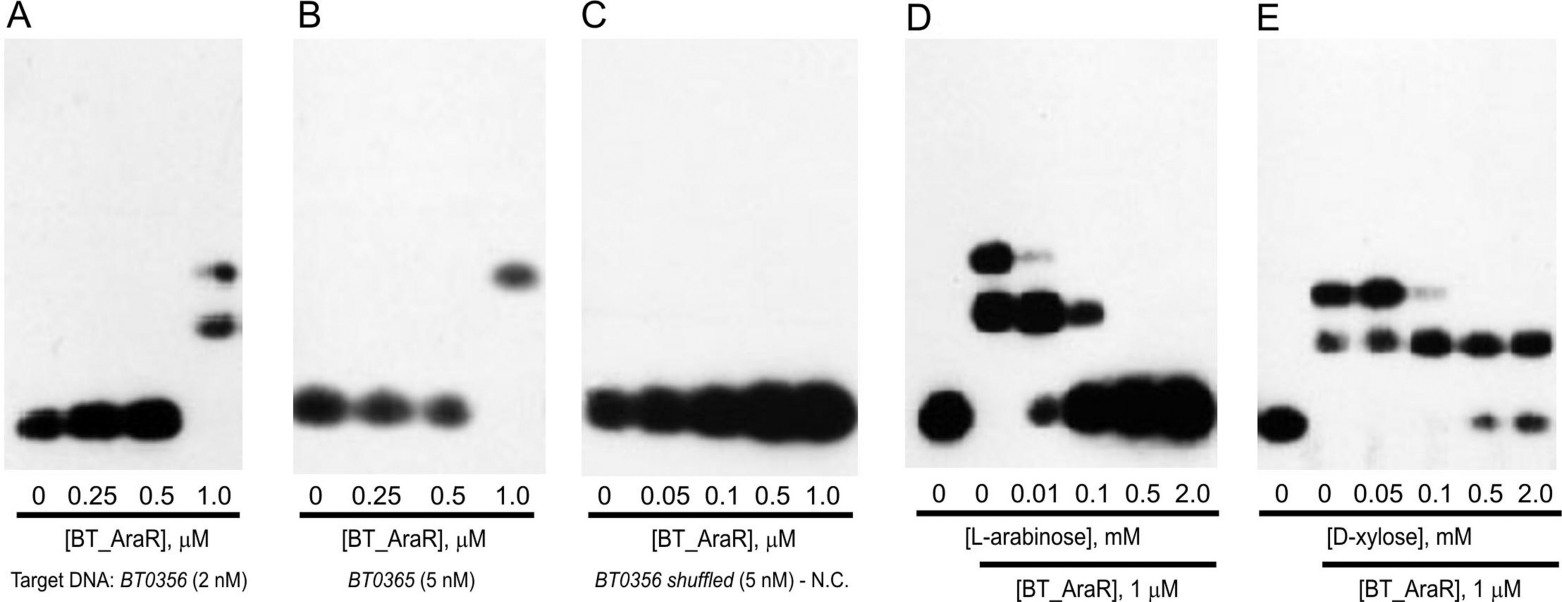
EMSA with BtAraR protein and its target DNA operators. (**A**–**C**) Titration of BtAraR protein for binding to DNA fragments from the BT0356 and BT0365 genes and the negative control (N.C.), which is a DNA fragment containing the shuffled sequence of BT0356. (**D**–**E**) Influence of sugar effectors on the formation of the BtAraR–DNA complex. The BtAraR protein (1 μM) and the BT0356 DNA fragment (0.2 nM) were incubated with increasing concentrations of L-arabinose and D-xylose in the incubation mixture (0.01–2 mM).

To validate the ability of BtAraR to specifically bind to the predicted DNA sites and to assess the role of possible effectors, we used EMSA. The BT0354 gene from *B. thetaiotaomicron* was cloned and overexpressed in *E. coli* and the recombinant BtAraR protein was purified to homogeneity. Binding of apo–BtAraR to synthetic biotin-labeled DNA fragments containing the predicted AraR sites at the AraR promoter regions of BT0356 and BT0365 genes was assessed. The 65-bp DNA fragment of the BT0356 promoter contains two tandem putative AraR sites, while the 41-bp DNA fragment of the BT0365 promoter represents a single AraR site. For both DNA fragments, mobility shifts of the DNA bands were observed in the presence of apo-BtAraR protein at concentrations of 1.0 μM (Figure 2A and B). For the BT0356 promoter two bands of protein/DNA complexes are observed, likely corresponding to one and two dimers of BtAraR bound to the promoter. The BT0365 promoter has a single AraR site and only one shifted band is observed. No gel shift of the DNA band was detected in the presence of control DNA (Figure 2C), confirming that apo-BtAraR recognizes specific DNA operator sequences.

In the second EMSA experiment, interaction between the BT0356 DNA promoter (containing two tandem AraR sites) and the BtAraR protein was assessed in the presence of various concentrations of L-arabinose (Figure 2D). The addition of 0.01 mM of L-arabinose significantly impaired the interaction between BtAraR and the promoter region, whereas the protein–DNA complex was completely abolished at 0.5 mM of the effector. We also tested another pentose sugar, D-xylose, for its ability to disrupt BtAraR binding to DNA. At D-xylose concentrations up to 2 mM, BtAraR still was able to bind to its cognate DNA promoter, although at 0.5 mM or larger concentration, D-xylose partially impaired the formation of the protein–DNA complex (Figure 2E). These results suggest that L-arabinose but not D-xylose is a specific sugar effector that regulates BtAraR DNA-binding activity.

### Structure determination and overall structure of BtAraR

We have solved four BtAraR structures by the SAD method. These include two apo–BtAraR forms, a complex with a specific DNA and a complex with the L-arabinose effector. The apo-protein crystallized in two different space groups: R3 rhombohedral (apo-1–BtAraR) and I-centered cubic, I2_1_3 (apo-2–BtAraR). In the apo-1–BtAraR structure, two dimer molecules are located in an asymmetric unit and the structure was refined to R/R_free_ of 0.195/0.233 at 2.35 Å resolution. The apo-2–BtAraR structure was refined to R/R_free_ of 0.183/0.222 at 2.56 Å resolution with one monomer in an asymmetric unit. The BtAraR–DNA complex crystallized in P23 cubic space group. One protein dimer and one DNA duplex are found in the asymmetric unit. The model was refined to R/R_free_ of 0.200/0.230 at 3.06 Å resolution. The structure of the ligand-bound form was refined to R/R_free_ of 0.164/0.220 and refined to 1.95 Å resolution with one dimer present in the asymmetric unit. All structures show acceptable r.m.s. deviation from ideal geometry and reasonable clash score from Morobity (43). The details of refinement statistics are in Table 1.

**Figure 3.**
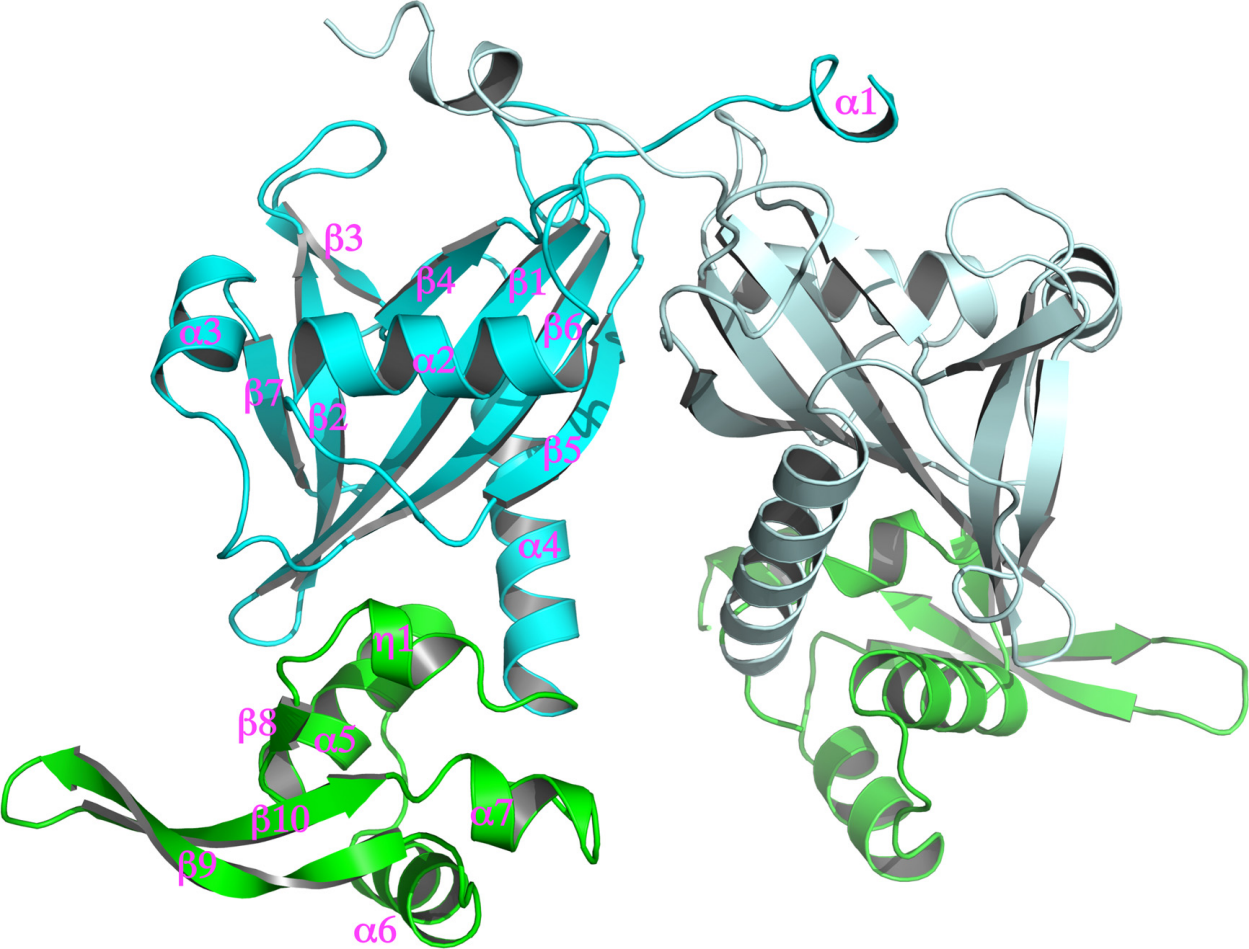
Ribbon diagram of dimeric structure of BtAraR. The N-terminal domain is colored as cyan, the C-terminal domain is colored as green. Molecule B is represented as paled color. The secondary structures are labeled.

The overall structure of BtAraR is quite similar to the previously solved NrtR protein (29% sequence identity), an ADP-ribose-dependent transcriptional regulator from *S. oneidensis* (17). The biological unit appears to be a dimer while a monomer is composed of two domains. The N-terminal domain (residues 1–148, Nudix domain) shows a Nudix hydrolase fold containing three α-helices and a seven-stranded β sheet along with an extra N-terminal helix of less than a turn. The C-terminal domain (residues 149–225) is a winged helix-turn-helix (wHTH) DNA-binding domain that has a protruding β-hairpin as a wing, and it is predicted to bind the major groove surface of the DNA using the HTH motif and the minor groove surface using the wing (Figure 3; Supplementary Figures S1 and S2).

The two apo structures are almost identical with an r.m.s deviation between the two structures of 0.42 Å. Interestingly, the apo–BtAraR proteins are more similar to the structure of AraR in complex with DNA than to the protein in complex with L-arabinose. An r.m.s. deviation between apo-1–BtAraR and the BtAraR–DNA complex is 1.13 Å, while that between apo-1–BtAraR and the L-arabinose-bound complex is 4.54 Å. The largest differences between the apo forms and the BtAraR–DNA complex are observed in the wing of the HTH motif. In the complex with DNA, the AraR β-hairpin loop, which includes two positively charged residues Lys204 and Arg205, moves toward the minor groove of the DNA. This is not observed in the apo structures. The Cα of Lys204 is moved about 4.2 Å and the Cα of Arg205 is moved about 6.1 Å toward the minor groove of the DNA (Figure 5C). In the L-arabinose-bound form, the C-terminal domains of the dimer are closer to each other as compared to the apo forms or the DNA-bound form. The details of these differences are discussed below.

In the L-arabinose-bound structure, two subunits are slightly different; r.m.s. deviation between molecule A and B in the L-arabinose-bound structure is 1.1 Å while that of the apo and the DNA-bound forms is only around 0.4 Å. The largest differences are observed for residues 103–107. These residues form an α-helix in molecule A but 3_10_ helices in molecule B. The other parts showing large differences in the L-arabinose-bound dimer are residues around Pro150 at the beginning of C-terminal domain. The C-terminal residues 220–225 in molecule B are close to molecule A and an electron density map is visible for this region, while in molecule A residues 219–225 are disordered. Moreover, molecules A and B also show significant difference in B-factors. Although B-factor profiles for molecules A and B are quite similar, the mean B-factor for molecule B is nearly double (41.8 Å^2^) that of molecule A (24.8 Å^2^).

The structures of BtAraR were compared with those of SoNrtR (Supplementary Figure S3). The r.m.s. deviation of the apo-forms of both regulators is 2.1 Å. The r.m.s. deviation between the ligand-binding domains of the L-arabinose-bound BtAraR and the ADP-ribose-bound SoNrtR is 1.7 Å. The r.m.s. deviation between the two overall protein structures of the DNA-bound forms is 2.2 Å. These two protein structures are similar despite the low sequence identity and different ligand specificity. The notable difference between the Nudix domains of BtAraR and SoNrtR (r.m.s.d. around 4.3 Å) was detected in the region around the α3 helix, which is presumably involved in a conformational change between the apo- and L-arabinose-bound forms of the BtAraR protein. The details of this structural change are discussed below.

### L-arabinose binding site

The L-arabinose binding site is located in the N-terminal Nudix domain and is formed by the β-sheet and the loop between β7 and α4, as found in other Nudix hydrolases. However, the BtAraR ligand-binding cleft is smaller than in other Nudix hydrolases. The N-terminus of BtAraR is stretched out to the adjacent monomer, partially covering the ligand-binding cleft typically occupied by a nucleotide moiety in other Nudix hydrolases. In the SoNrtR structure, the N-terminus is also extended toward the adjacent monomer, but it is located further away from the nucleotide-binding cleft, leaving enough space for an ADP moiety of ADP-ribose (Supplementary Figure S3B). Similar crossover of the N-terminus is also found in other Nudix hydrolases such as DR1025 from *Deinococcus radiodurans* (44).

**Figure 4.**
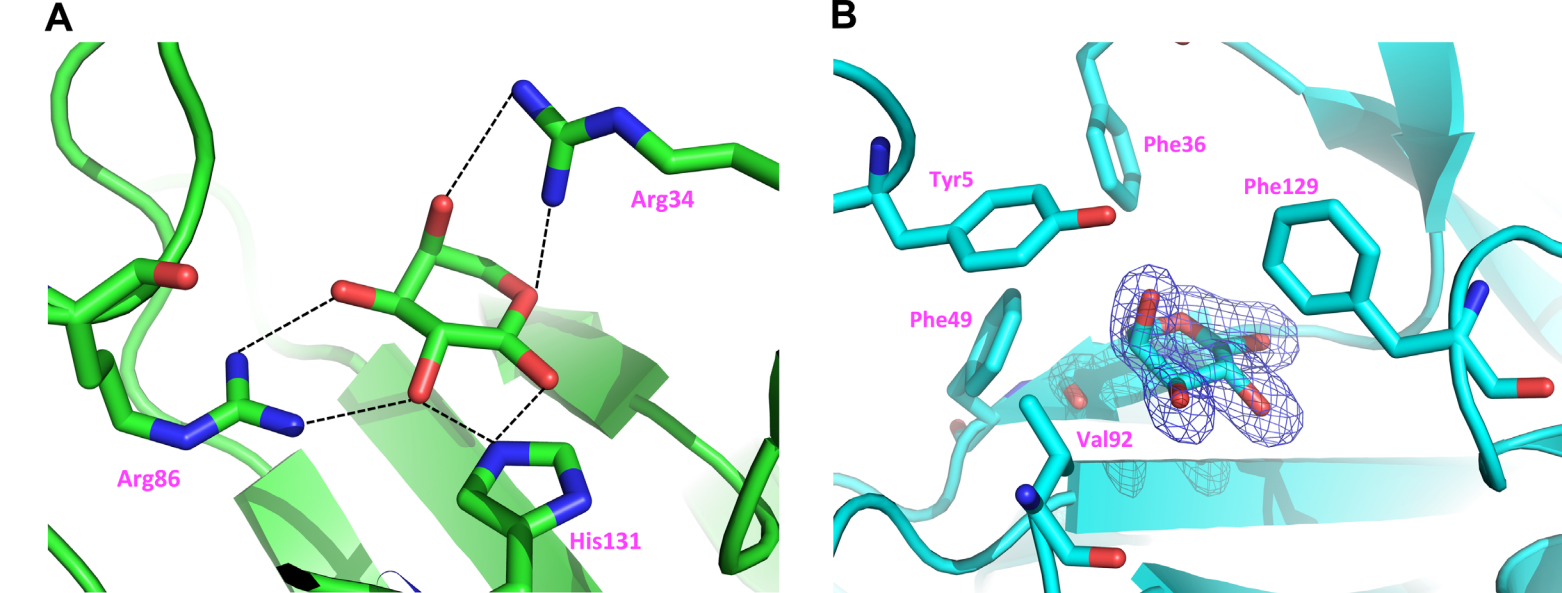
L-Arabinose and arabinose-binding pocket in BtAraR. The residues interacting with L-arabinose are represented as stick model and labeled. (**A**) The hydrogen network around L-arabinose. Hydrogen bonds are indicated as black dotted lines. (**B**) The hydrophobic residues around L-arabinose. Omit map for L-arabinose is represented as blue mesh. The contour level of omit map is 5σ. The hydrophobic residues interacting with L-arabinose are labeled.

All four hydroxyl groups of L-arabinose are involved in a hydrogen bond network with side chains of Arg34, Arg86 and His131 (Figure 4A). These arabinose-binding residues are fully conserved in all AraR orthologs in other *Bacteroides* and *Prevotella* spp. (Supplementary Figure S1), suggesting that the specificity toward the L-arabinose effector is conserved. Compared to the previously reported SoNrtR protein, these residues, except Arg34, are also spatially well conserved. In BtAraR, Arg34 makes a hydrogen bond with O4 of L-arabinose, while corresponding residue Arg41 in the SoNrtR–ADP-ribose complex makes a hydrogen bond with the phosphate oxygen of ADP-ribose. In BtAraR, residues Tyr5, Phe36, Gly47, Gly48, Phe49, Val92 and Phe129 contribute to hydrophobic interactions with the ligand. Among these, Tyr5, Phe36, Phe49 and Val92 provide a hydrophobic environment for C4 and C5 of L-arabinose, and Phe129 points toward C2 of L-arabinose, defining stereo-selectivity of this atom (Figure 4B). Most of these residues contributing to hydrophobic interactions are conserved in all AraR orthologs, with the exception of Phe36 and Phe129 that are substituted in the AraR orthologs from *Prevotella* species with methionine and serine, respectively (Supplementary Figure S1).

Several mutants of BtAraR (R34N, F49Q and V92D) were constructed to investigate a role of these residues in L-arabinose binding. The activity of these mutants was evaluated using the EMSA assay (Supplementary Figure S4). R34N and F49Q mutants still bound DNA when L-arabinose was added at 0.5 mM concentration. The residue Phe49 contributes a hydrophobic environment for C5 atom of L-arabinose; therefore, the mutation of phenylalanine to polar glutamine may disturb the hydrophobic environment and reduce binding of L-arabinose. The V92D mutant does not bind DNA. The residue Val92 is located near the dimer interface, and the mutant V92D does not form dimers as observed by dynamic light scattering (Supplementary Figure S5). Therefore, we can assume that only AraR dimers are capable of binding to the specific promoter region.

As described above, the binding cleft for a nucleotide moiety that is present in other Nudix domains is missing in the structure of BtAraR. Here we compared that region with SoNrtR. In the SoNrtR structure, the residue Arg98 makes hydrogen bonds with O5D and O1B of ADP-ribose. This residue is replaced with Gly89 in BtAraR and the space for the arginine side chain was filled with the side chain of Tyr5. The corresponding residue for Tyr5 in SoNrtR is Tyr10, which makes hydrogen bonds with N6 and N7 of adenine base. Residue Asn8 makes a hydrogen bond with Gln51 of the adjacent molecule, then the N-terminal residues get close. The corresponding residue in SoNrtR, Phe15, makes a π interaction with adenine base. Residue Lys76 of SoNrtR, making a hydrogen bond with α-phosphate of ADP, is substituted with Leu67 in BtAraR. The residue Asn43 of SoNrtR, making another hydrogen bond with α-phosphate of ADP, was replaced with Phe36 in BtAraR, and the side chain of Phe36 is located in the space of the phosphate group. Therefore, the spaces for phosphates and the base are all filled with protein side chains in BtAraR.

The most important change near the ligand-binding cleft that occurs upon L-arabinose binding is the movement of Arg34 about 3.0 Å along with Phe36 toward the L-arabinose. The Arg34 guanidinium moiety hydrogen bonds with O4, which pulls the loop between β2 and β3 about 3.5 Å into the binding pocket. This conformational change makes the binding pocket compact and thus more selective to ligands. Consequently, the α2 is pulled away from the C-terminal domain and α3 is pushed down toward the C-terminal domain. With this rearrangement, Tyr74 located between α2 and β5 is liberated from the hydrophobic core formed between the two domains, making this part more flexible but less stable, as evident by the conversion of a 3_10_ helix of residues 152–155 into a loop. It appears that all the conformational changes triggered by the L-arabinose binding make the dimer tighter but less favorable to bind DNA.

### Structure of specific BtAraR–DNA complex

**Figure 5.**
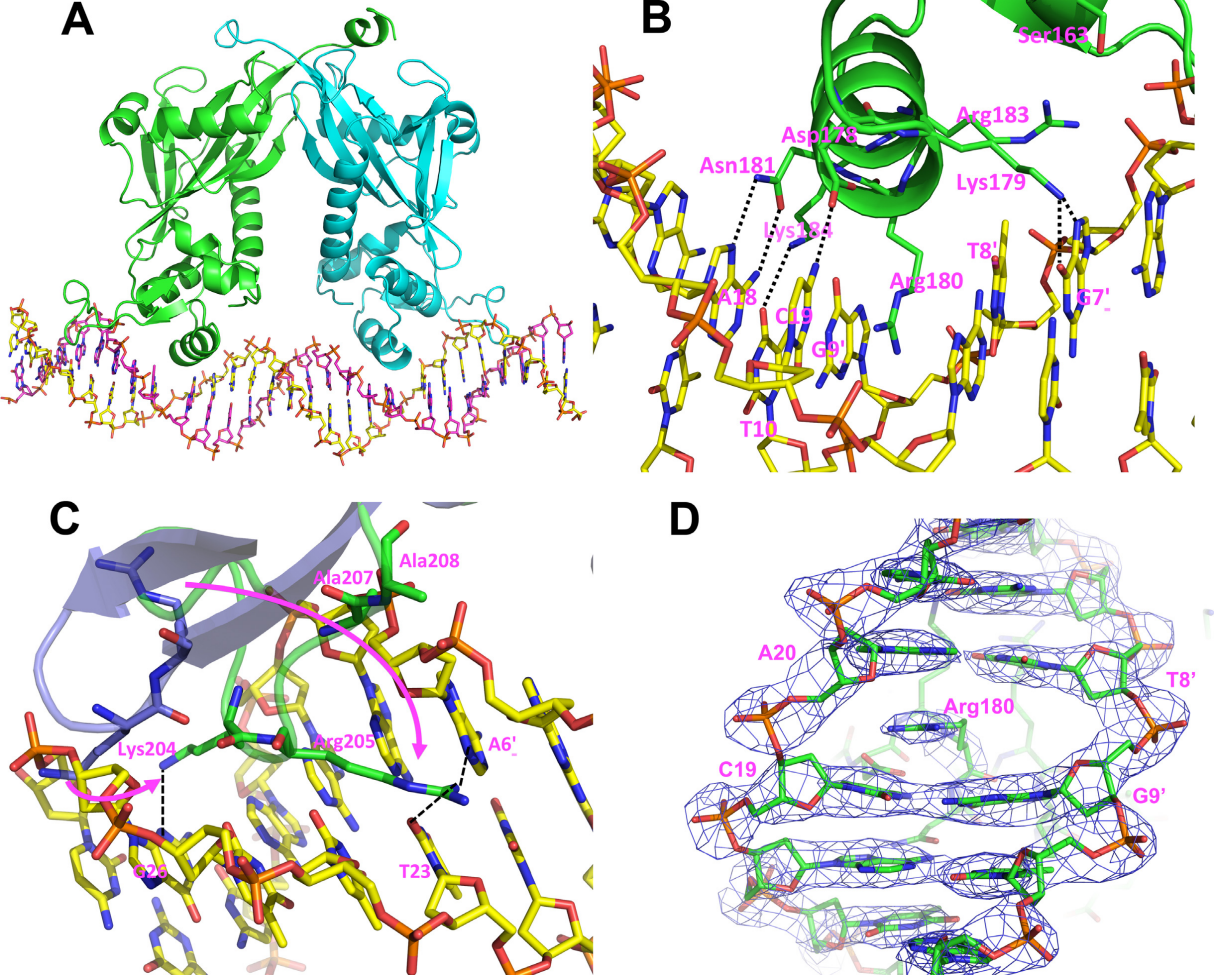
Protein–DNA interaction in BtAraR–DNA complex. (**A**) Overall structure of BtAraR in complex with DNA double strands. BtAraR is represented as ribbon diagram with molecule A colored as green, molecule B as blue. Specific double stranded DNA is represented as stick model with strand C colored as yellow, strand D as purple. (**B**) The interactions in major groove. The DNA duplex is represented as stick model with carbon color of yellow. The protein molecule is represented with ribbon model while the residues interacting with DNA are stick model. The residues making interactions are all labeled. The hydrogen bonds are indicated as black dotted lines. (**C**) The interactions in minor groove. The protruding wing in the C-terminal domain is fitted to the minor groove of the DNA duplex. The apo form of BtAraR is represented as dark blue while the protein in complex with DNA is represented as green. The DNA-interacting residues are shown as stick model and the DNA model is represented as stick model with carbon color of yellow. The arrows show the adaptation of Lys204 and Arg205 for fitting on the DNA minor groove. (**D**) Electron density map around side chain of Arg180. The DNA duplex and protein are represented as stick model. Electron density map (2Fo–Fc) contour level is 1.7 σ.

The overall protein structure of the BtAraR–DNA complex is similar to the apo-protein structure (r.m.s. deviation is 1.13 Å), while it is quite distinct from the structure of BtAraR with bound L-arabinose (r.m.s. deviation is 4.28 Å). The 27-bp DNA duplex shows slight distortion from B-DNA conformation with 7.5° bending in the middle, causing both ends of the DNA to move toward the protein (Figure 5A).

The C-terminal wHTH domain is well known to recognize and bind double stranded B-DNA. The HTH motif interacts with the major groove surface (Figure 5B) and the β-hairpin wing interacts with the minor groove (Figure 5C). Although the sequence of DNA is not fully palindromic, the interaction of protein with DNA is quite two-fold symmetric. The specific interaction between BtAraR and the 27-bp DNA duplex includes several direct hydrogen bonds, water-mediated hydrogen bonds, hydrophobic and van der Waals contacts. The protein dimer covers 25 bp of the duplex. It also involves both protein and DNA deformation to fit each other specifically. Arg180 shows a unique feature for interaction with DNA. The side-chain of Arg180 with its guanidinium moiety deeply penetrates the DNA duplex and intercalates between bases C19 and A20 in the major groove (Figure 5D). This interaction is identical for both protein monomers. The planar guanidinium moiety perfectly stacks with neighboring bases, and the distances between the planes are 3.1–3.3 Å for chain A and 3.2–3.6 Å for chain B, quite comparable to typical base pair stacking distance in B-DNA. One similar case is observed in the DNA complex structure of PD-(D/E)XK type II restriction endonuclease ThaI (PDB entry 3NDH (45)). However, in this structure, methionine rather than arginine was intercalating and showed less efficient stacking.

Residues Lys179, Asn181 and Lys184 of helix α6 in the HTH motif make hydrogen bonds with edges of DNA bases in the major groove. Lys179 makes strong direct hydrogen bonds with N7 and O6 in G7 in both monomers. Asn181 makes a similar but longer hydrogen bond with A18. A ND2 of Asn181 makes a hydrogen bond with N7 of A18 and OD1 of Asn181 makes a hydrogen bond with N6 amino group of A18. Lys184 forms an even longer hydrogen bond with T10. In protein chain A and DNA strand C, hydrogen bond distance between NZ of Lys184 and O4 of T10 is 3.2 Å, while that of chain B and strand D is only 3.7 Å, suggesting that there is some adjustment of the two molecules during binding (or initial interaction involves water-mediated contacts). In the minor groove, Lys204 and Arg205 in the β-hairpin wing are involved in direct hydrogen bonding with DNA bases. NZ of Lys204 makes a hydrogen bond with N3 of G26. NH1 of Arg205 makes a hydrogen bond with N3 of residue A6 in strand C (or atom N3 of residue G6 in strand D). Again, in chain A, Arg205 approaches the DNA closer than in chain B; NH1 interacts with O2 of T23 with a hydrogen bond length of 3.07 Å in chain A and 3.43 Å in chain B. The interaction of these residues in the major and minor grooves defines GTGT DNA sequence motif. We expect some water-mediated hydrogen bonding to occur as well, but at this resolution, we cannot confidently model bridging water molecules in water-mediated hydrogen bonds. Other than hydrogen bonds, residues Ser163, Asp178, Arg183, Arg180 and Lys184 make van der Waals contacts in the major groove, while Lys200, Ala207 and Ala208 make van der Waals contacts in the minor groove. Among these, Ser163, Arg183 and Ala208 make contact with atoms in the phosphates of A6 and G7 with chain A, G6 and G7 with chain B. Lys200 in chain B makes contact with G26 in strand C. The details on hydrogen bonding networks and van der Waals interaction contacts between protein and DNA molecules are summarized in Table 2 and Supplementary Figure S6. All identified DNA-contacting residues (Lys179, Arg180, Asn181, Lys184, Lys204 and Arg205) are absolutely conserved in all AraR orthologs among the *Bacteroidetes* phylum, suggesting conservation of their DNA-binding sites (Supplementary Figure S1).

**Table 2.** Specific DNA–protein interactions

No	Base	Interaction	Dist.	No	Base		Dist.
C1	G			D27	C		
C2	C			D26	G	**N3–A 204 Lys NZ**	2.98
C3	A			D25	T		
C4	A			D24	T		
C5	A			D23	T	**O2–A:205 Arg NH1**	3.07
C6	A	**N3–A: 205 Arg NH1**	2.76	D22	T		
		vdW A:163 Ser					
		vdW A:207 Ala					
C7	G	**N7–A: 179 Lys NZ**	2.79	D21	C		
		**O6–A: 179 Lys NZ**	2.82				
		vdW A: 183 Arg					
		vdW A:208 Ala					
C8	T	vdW A: 180 Arg		D20	A		
C9	G	vdW A: 180 Arg		D19	C	vdW A:178Asp	
						vdW A: 180 Arg	
C10	T	**O4–A:184 Lys NZ**	3.20	D18	A	**N6–A 181 Asn OD1**	2.97
						**N7–A 181Asn ND2**	3.15
C11	T			D17	A		
C12	A			D16	T		
C13	C			D15	G		
C14	T			D14	A		
C15	T			D13	A		
C16	T			D12	A		
C17	T			D11	A		
C18	A	**N6–B: 181 Asn OD1**	3.04	D10	T		
		**N7–B:181 Asn ND2**	3.06				
C19	C	**N4–B: 178 Asp OD2**	3.18	D9	G	vdW B:180Arg	
		vdW B:180 Arg					
C20	A			D8	T	vdW B: 180 Arg	
C21	C			D7	G	**N7–B: 179 Lys NZ**	2.63
						**O6–B: 179 Lys NZ**	2.91
						vdW B: 183 Arg	
						vdW B: 208 Ala	
C22	C			D6	G	vdW B:163 Ser	
						vdW B:207 Ala	
						**N3–B:Arg 205 NH1**	2.99
C23	C			D5	G		
C24	A			D4	T		
C25	T	vdW B:200 Lys		D3	A		
C26	G	**N3–B:204 Lys NZ**	2.93	D2	C		
C27	C			D1	G		

The bold letters mean hydrogen bond and vdW means van-der-Waals interactions.

When DNA parameters are calculated using the DNA analysis program Curves+ (37), most base pair parameters show values similar to those of the ideal B-DNA. However, the stacking of base pairs T8-A20′/G9-C19′ and C19-G9′/A20-T8′ indicates atypical conformation due to the aforementioned Arg180 intercalation between these two base pair steps. The rise of steps for T8-A20′ to G9 -C19′ and C19-G9′ to A20-T8′ base pairs are 6.96 Å and 7.24 Å, respectively. These values are twice the typical rise value for B-DNA. The DNA duplex can be extended by symmetry-related DNA molecules in crystal lattice and forms a continuous double helix. In the crystal G1-C27′ base pair stacks with C27-G1′ base pair of a symmetry-related molecule. However, their twist angle is opposite to normal base pair stacking. As a consequence, G1-C27′ base pair makes zero twist angle with the second G26-C2’ base pair of a symmetry-related molecule. With the exception of later interactions, the stacking extends the double helix of DNA with normal B-DNA parameters including the DNA axis and base–base distances.

### Arg180 is intercalated into base pair stacking in DNA duplex

Arg180 shows a unique feature for interaction with DNA. This is the first reported case of amino acid intercalation into a DNA duplex that does not disrupt regular base stacking significantly. One similar case of amino acid intercalation, observed in the DNA complex structure of PD-(D/E)XK type II restriction endonuclease ThaI (PDB entry: 3NDH (45)), showed that the methionine side chain intercalation interrupted regular stacking of base pairs, causing unwinding of the DNA. However, in the case of BtAraR, the intercalation of the side chain of arginine into the DNA duplex causes parallel translation of base pair, so the inter-base pair parameters, other than rise, are normal. This intercalation causes enlarged pitch of double stranded DNA in BtAraR. The distance between D12:P and D22:P is 38.6 Å, while in the SoNrtR–DNA complex it is 33.1 Å (Supplementary Figure S3C). As the pitch expands, the distance between two α6 recognition helices, which penetrate the major groove of DNA, is about 28.4 Å, while that in the SoNrtR structure is shorter (23.4 Å). This striking deformation seems to be contributing to the specificity of the BtAraR–DNA recognition. However, the point mutation R180K does not abolish DNA binding, although less DNA was shifted in EMSA with 1 μM of the mutated protein when compared to the similar experiment with the wild-type BtAraR (data not shown). Given its size, bond electron distribution, and flexibility, the lysine residue is unlikely to make the a similar intercalation into the DNA duplex. Quite often, specific interaction in protein-nucleic acid recognition is over-determined in most biological systems, and in cases of a few failures in specific interaction, the overall specificity is still maintained with affordable attenuation. We attempted to obtain the structure of R180K and visualize the interaction of the lysine residue with DNA, but thus far the co-crystallization efforts with the DNA target have failed.

### Helix-turn-helix domain is rotating upon L-arabinose binding

L-arabinose is an effector molecule that reduces affinity of BtAraR for a specific DNA target. The previously reported structures of the Nudix transcriptional factor SoNrtR do not include the full-length structure in complex with ligand molecule. Only the ligand-binding domain was solved in complex with ligand. Therefore, there is no information about conformational changes caused by ligand-binding leading to a lower affinity of the effector–Nudix complex toward a DNA target. Here, we determined the structures of full-length apo-protein, its complex with a DNA target, and its complex with the L-arabinose effector. These structures explain how BtAraR DNA-binding affinity is lowered upon sugar binding.

**Figure 6.**
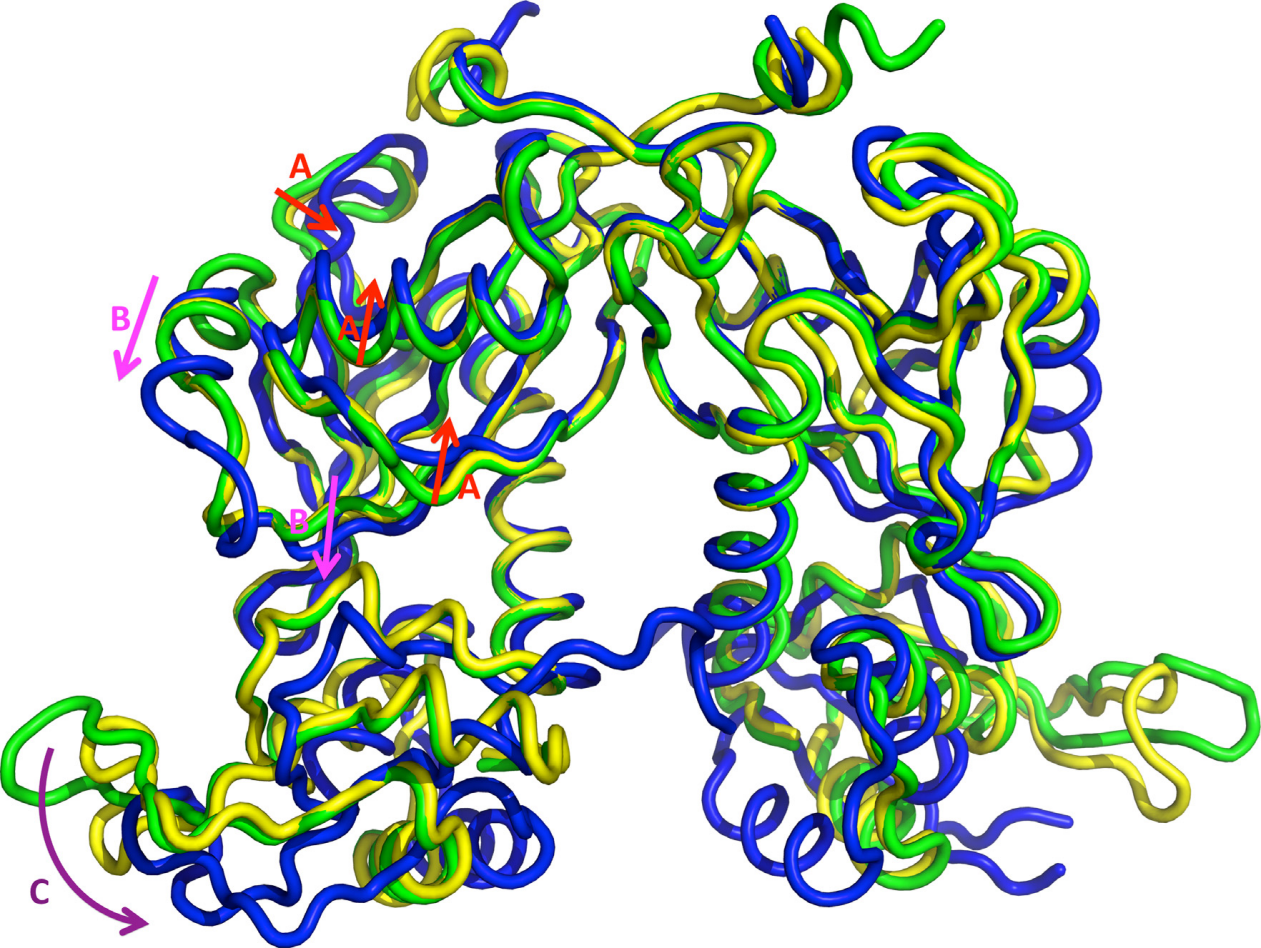
Structure comparison of apo, DNA-complex and L-arabinose-bound forms of BtAraR. The apo-protein structure is colored in green, the protein structure in DNA complex is colored in yellow and the L-arabinose-bound protein structure is in blue. (**A**) Red arrows show the movement of the L-arabinose-binding site to make this cleft more compact. (**B**) Pink arrows show the movement of α3 and β6. (**C**) Purple arrow shows the rotation of the C-terminal domain.

One of the largest conformational changes in the Nudix domain upon L-arabinose binding occurs in the loop between β2 and β3 strands. The strong interactions of Arg34 with L-arabinose pull the loop about 3.5 Å into the binding pocket. This change is concurred with moving of the surrounding secondary structure elements (Figure 6A) (shifting β2 and β3 strands, moving β4 and α2 closer to the L-arabinose binding cleft). The region around helix α3 also shows large movement (Figure 6B). This region has been described previously as the ‘Nudix switch’. It corresponds to the most flexible loop, including key catalytic residues of Nudix hydrolases (17,46–47). BtAraR does not have Nudix catalytic residues but instead, this region assumes an α helix (named α3). This helix is moved toward the C-terminal domain ∼4.5 Å along with a part of strand β6 (∼1.5 Å). These movements in the Nudix domain cause rotation of the entire C-terminal domain by about 15° (Figure 6C). The connection between the N-terminal and C-terminal domains, helix α4, serves as a hinge for this movement. As a result of the C-terminal domain's rotation, they get closer in the dimer. The helix α6 of the HTH motif moves around 8 Å and the protruding β-hairpin wing moves about 9 Å as compared with the apo structure. These adjustments change the distance between the two α6 helices from ∼30 Å in apo-BtAraR and in the complex with DNA, to ∼14 Å in the complex with L-arabinose. The separation of two wHTH motifs in the apo form allows a good fit into the major grooves of the DNA duplex, but this is no longer possible after the wHTH motif rotation in the L-arabinose-bound form. Therefore, the affinity for DNA is significantly reduced and the operon is upregulated.

### Concluding remarks

In summary, by combining the comparative genomics-based regulon reconstruction with the focused biochemical and structural characterization, we identified a novel arabinose-responsive transcription factor in the *Bacteroides* involved in utilization of arabinose and arabinose-containing oligosaccharides. This regulator, termed BtAraR, belongs to the NrtR family of transcriptional regulators with unusually divergent functional specificities (from NAD to pentoses). Cognate AraR-binding sites predicted in the *B. thetaiotaomicron* genome were validated by DNA binding assays; however, the exact determinants of protein–DNA specificity remain to be investigated via mutagenesis of DNA sites. We determined the structures of full-length apo–BtAraR protein, its complex with a specific DNA duplex, and its complex with the L-arabinose effector. The solved structures provide a first view of the structural mechanism underlying the regulatory function of the L-arabinose-binding Nudix domain. The movement of several secondary structure elements upon effector binding leads to domain rotation and causes major changes in the dimer conformation. These changes affect the orientation of the wHTH domains, leading to disruption of the protein–DNA interaction. Conservation of the AraR residues involved in the interaction with L-arabinose and with the specific DNA sequences, as well as the conservation of DNA-binding motifs of AraR in other species, together suggest that all orthologs of AraR identified in the *Bacteroides* and *Prevotella* species retain their molecular function and specificity toward effector and DNA molecules. The obtained novel insights on the AraR regulons in the *Bacteroidetes* will facilitate reconstruction of associated metabolic pathways in diverse bacterial species from this phylum and will lead to improved functional predictions for the entire NrtR family.

## ACCESSION NUMBERS

The structures were deposited to PDB entry codes: 5BS6 (apo-1–BtAraR), 5DD4 (apo-2–BtAraR), 5DDG (in complex with DNA) and 5DEQ (L-arabinose-bound).

## Supplementary Material

SUPPLEMENTARY DATA
