# Supplementary material for: A novel transcriptional regulator of L-arabinose utilization in human gut bacteria
Source: Nucleic Acids Res. 2015 Oct 4;43(21):10546–59. doi: 10.1093/nar/gkv1005 (PMC4666351; doi:10.1093/nar/gkv1005)
Supplement: SUPPLEMENTARY DATA [file supp_43_21_10546__index.html]

A novel transcriptional regulator of L-arabinose utilization in human gut bacteria — SUPPLEMENTARY DATA 

# A novel transcriptional regulator of L-arabinose utilization in human gut bacteria

## SUPPLEMENTARY DATA

- SUPPLEMENTARY DATA
- SUPPLEMENTARY DATA
- SUPPLEMENTARY DATA
- SUPPLEMENTARY DATA
- SUPPLEMENTARY DATA
- SUPPLEMENTARY DATA
- SUPPLEMENTARY DATA
- SUPPLEMENTARY DATA
