# Supplementary material for: A novel transcriptional regulator of L-arabinose utilization in human gut bacteria
Source: Nucleic Acids Res. 2015 Oct 4;43(21):10546–59. doi: 10.1093/nar/gkv1005 (PMC4666351; doi:10.1093/nar/gkv1005)
Supplement: SUPPLEMENTARY DATA [file supp_gkv1005_nar-02513-h-2015-File008.doc]

**Supplemental Figures and Tables**

**Figure S1. Multiple alignment of AraR proteins from the NrtR family.** AraR orthologs are named by their cognate species name from the *Bacteroides* and *Prevotella* genera and are listed in Supplementary Table S1. The N-terminal Nudix-like ligand-binding domain and C-terminal wHTH DNA-binding domains are shown by yellow and pink bars, respectively. Secondary structure elements according to the BtAraR (BT0354) crystal structure are shown by zigzags (α-helices) and arrows (β-strands). Residues in the BtAraR protein involved in interaction with DNA and L-arabinose are shown by red arrows on the top of the alignment. The position of the Nudix signature motif, which is only partially conserved in AraR proteins, is also shown on the top.

**Figure S2. Sequence alignment of BtAraR, BtXylR, and SoNrtR with secondary structure.** Structure alignment and comparison were performed using ClustalW and ESPript 3.0 . The top line shows secondary structure elements of apo-BtAraR, the bottom line of each row shows that of apo-SoNrtR.

**Figure S3. Superposition of crystal structures of BtAraR and SoNrtR.** Ribbon diagram of the crystal structures of BtAraR and SoNrtR. BtAraR structures are colored as green, SoNrtR structures are colored as pale blue. **A. Superposition of apo forms.** PDB code for apo-SoNrtR is 3GZ5. **B. Superposition of proteins in complex with DNA duplex**. PDB code for SoNrtR in complex with DNA is 3GZ6. **C. Superposition of ligand-bound forms**. Ligand molecules are represented as stick model. PDB code for SoNrtR in complex with ADP-ribose is 3GZ8.

**Figure S4. EMSA with BtAraR mutants and *BT0356* DNA promoter fragment.** DNA concentration is 0.16 nM and protein concentration is 1.0 μM. L-arabinose was used as the effector. A control of DNA without protein was applied on lane 1.

**Figure S5.** **Size distribution profile of wild type (A) and V92D mutant (B).** The regularization plots were generated using DYNAMICS^TM^ Ver. 6.10.1.2 on dynamic light scattering device DynaPro Plate Reader (Wyatt Technology Corporation) **A**. Wild type BtAraR forms a stable dimer (60 kDa) as indicated in the profile (3.4 nm, poly-dispersity of 18.9, and 60 kDa). **B.** With the apparent size of 32 kDa, the V92D mutant fails to form a dimer (2.6 nm, poly-dispersity of 25.3, and 32 kDa).

**Figure S6. Schematic diagram of protein-DNA interaction in BtAraR-DNA complex.** Hydrogen bonds are indicated as orange arrows. Blue arrows indicate van der Waals interaction.

**Table S1. Candidate AraR binding sites and reconstructed regulons in the genomes of *Bacteroides* and *Prevotella* species.**
