# Supplementary figures and images for: A novel transcriptional regulator of L-arabinose utilization in human gut bacteria
Source: Nucleic Acids Res. 2015 Oct 4;43(21):10546–59. doi: 10.1093/nar/gkv1005 (PMC4666351; doi:10.1093/nar/gkv1005)

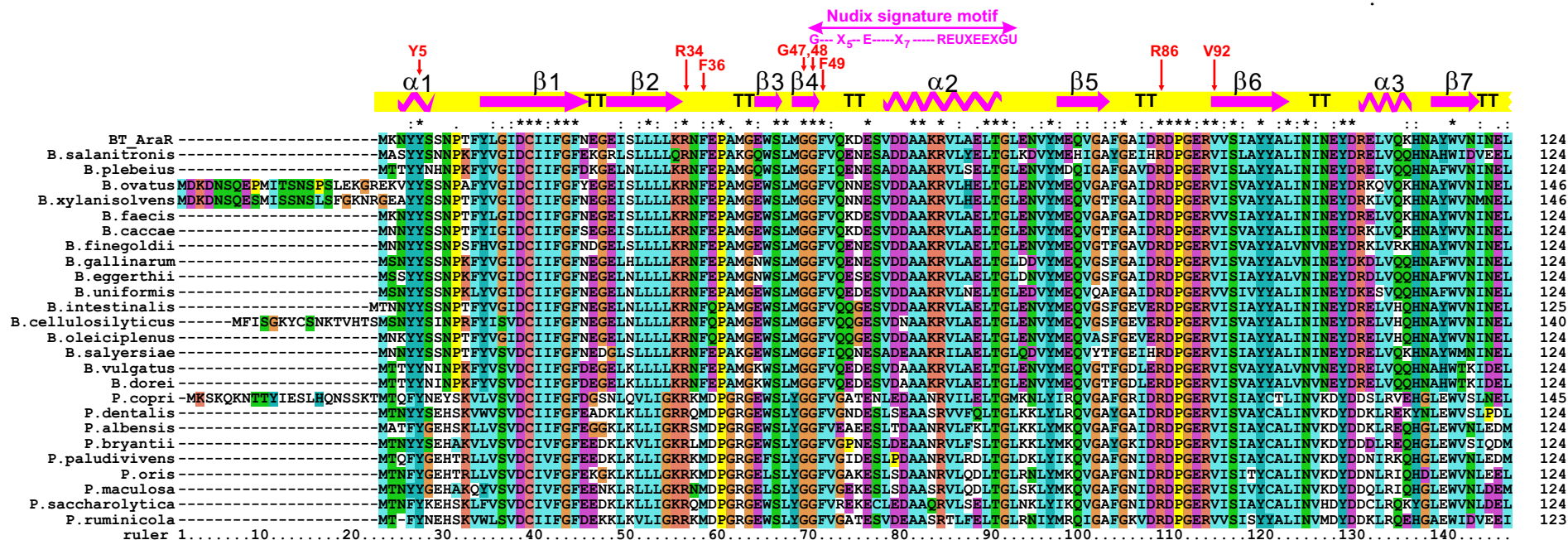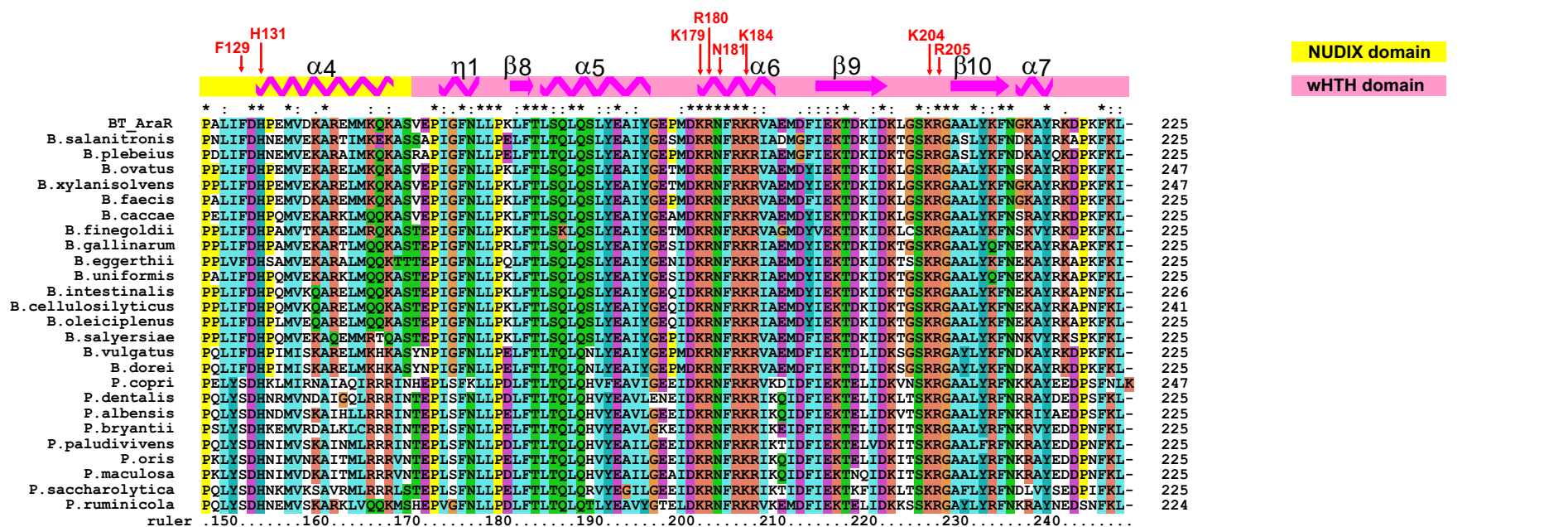

**NUDIX domain**

**wHTH domain**

Supplement: SUPPLEMENTARY DATA [file supp_gkv1005_nar-02513-h-2015-File009.pdf]

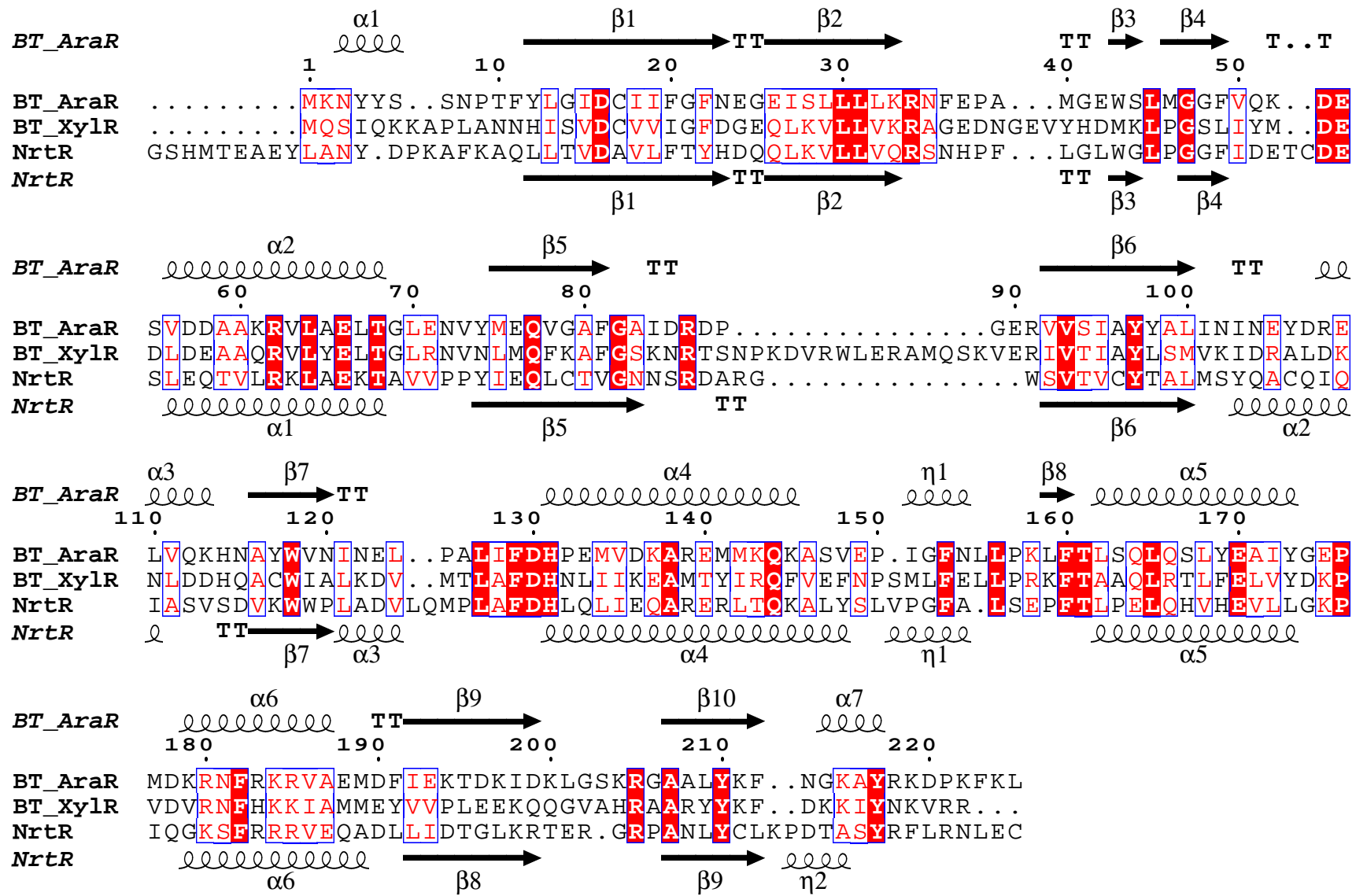

Supplement: SUPPLEMENTARY DATA [file supp_gkv1005_nar-02513-h-2015-File010.pdf]

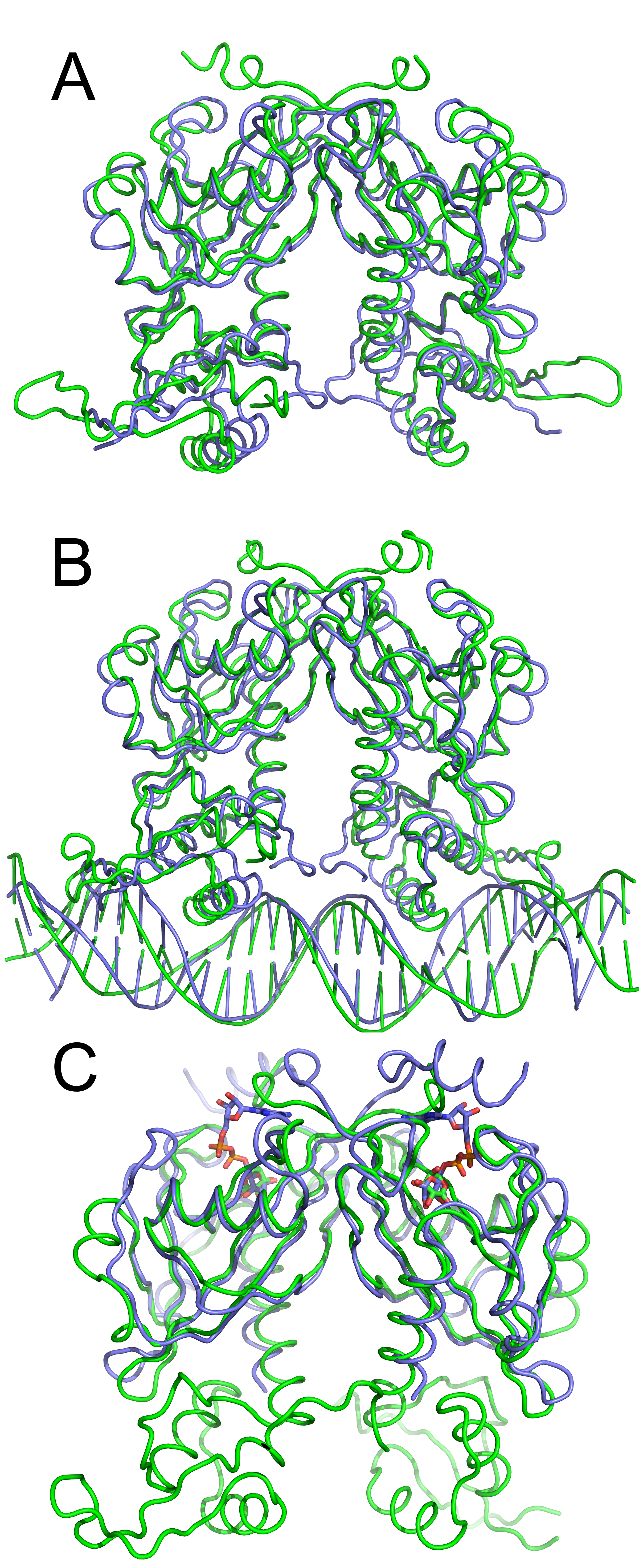

Supplement: SUPPLEMENTARY DATA [file supp_gkv1005_nar-02513-h-2015-File011.tif]

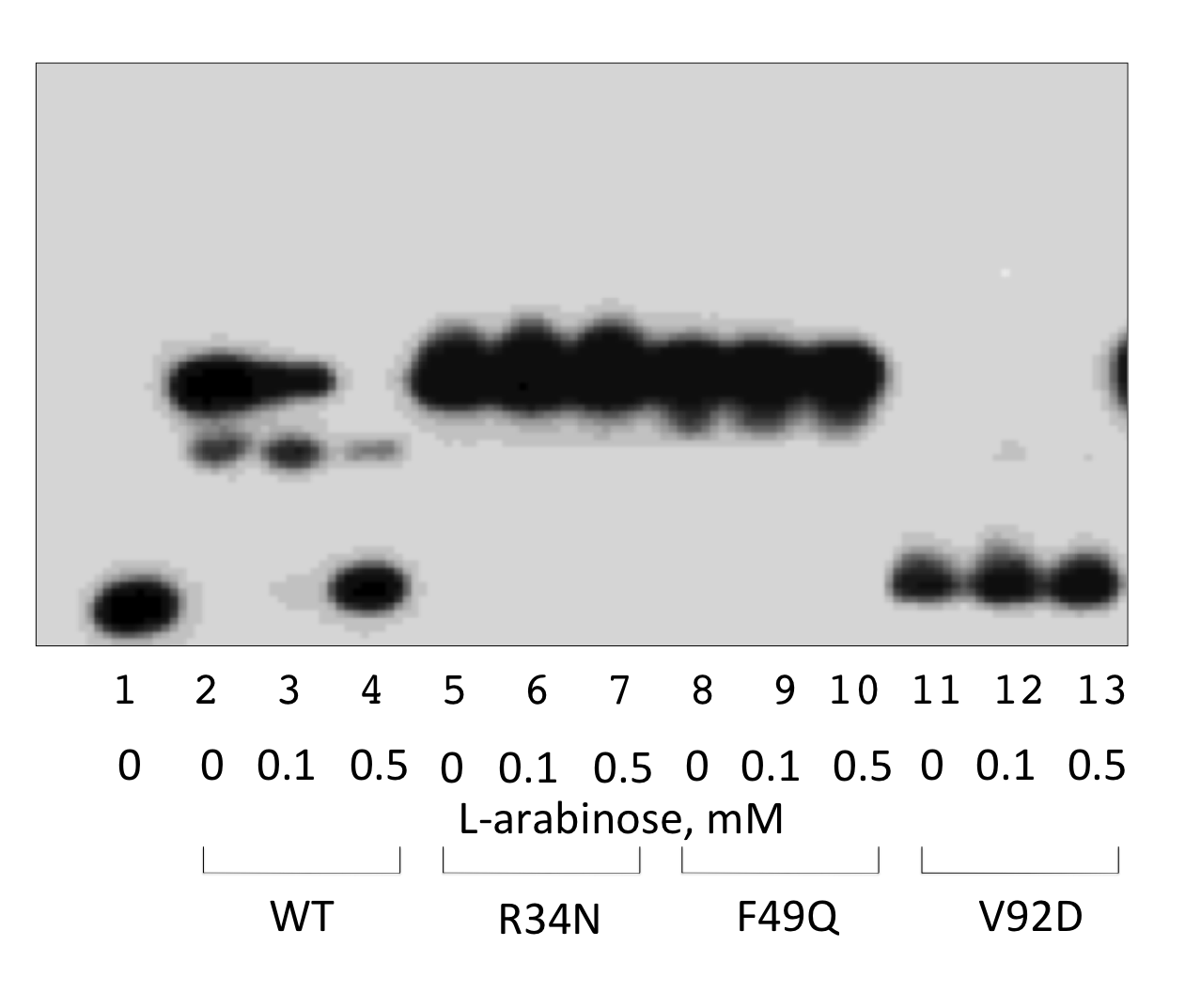

Supplement: SUPPLEMENTARY DATA [file supp_gkv1005_nar-02513-h-2015-File012.tif]

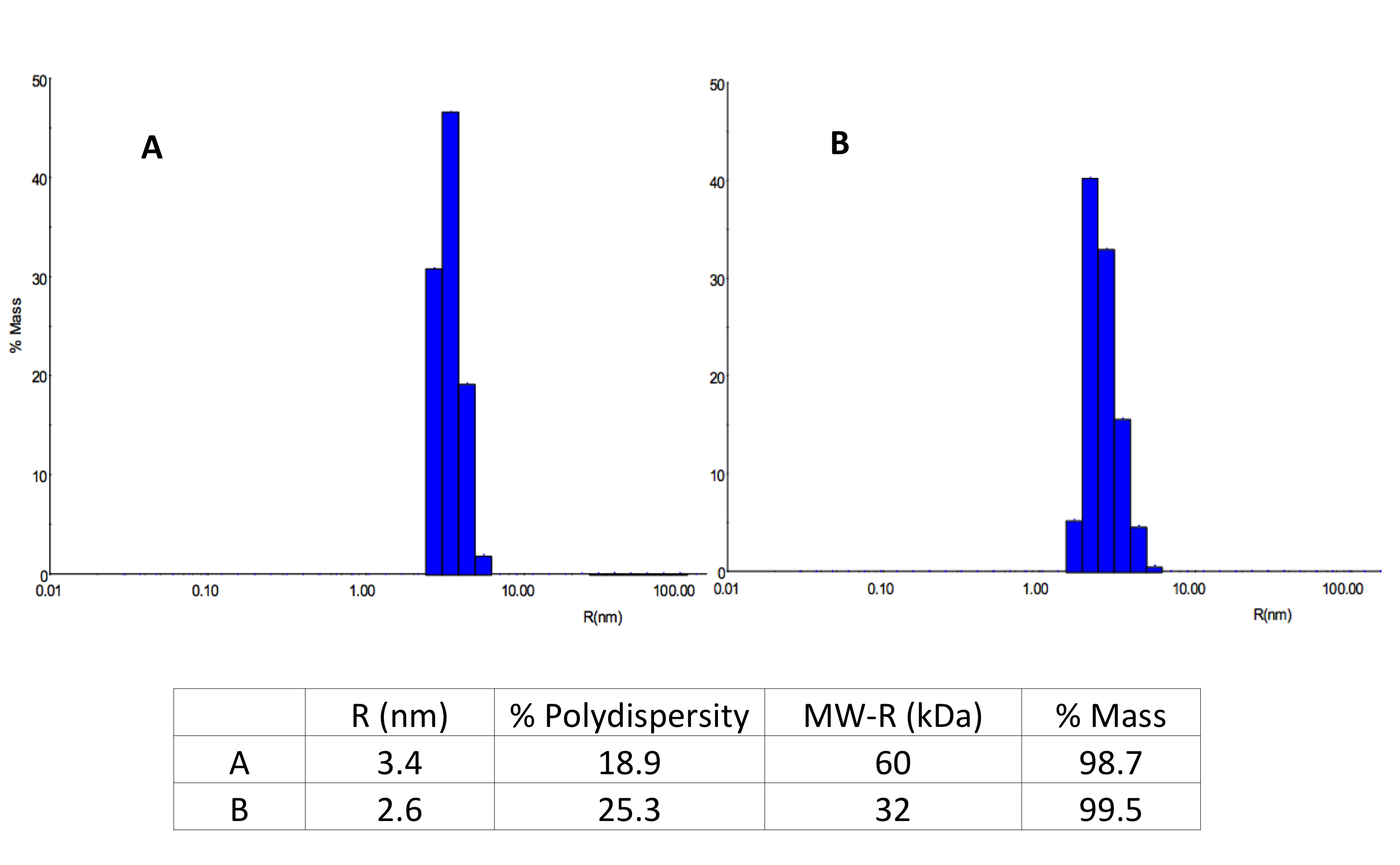

Supplement: SUPPLEMENTARY DATA [file supp_gkv1005_nar-02513-h-2015-File013.tif]

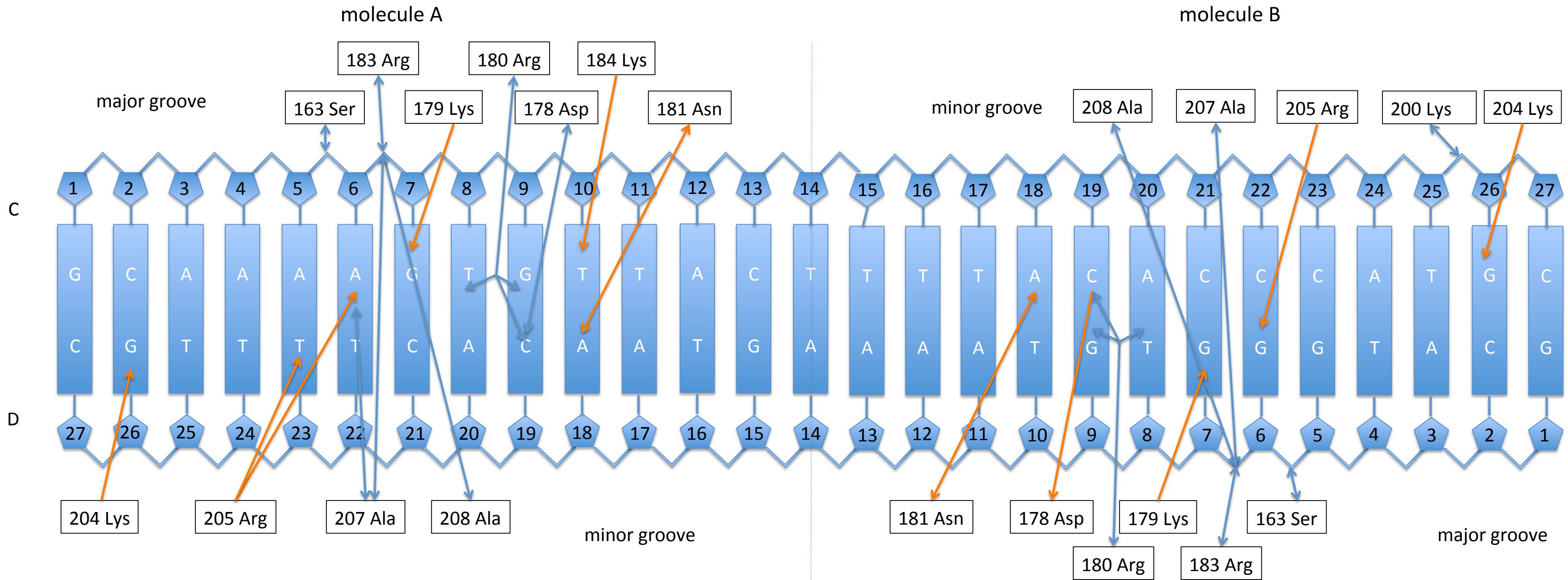

Supplement: SUPPLEMENTARY DATA [file supp_gkv1005_nar-02513-h-2015-File014.pdf]
