# Supplementary material for: A novel transcriptional regulator of L-arabinose utilization in human gut bacteria
Source: Nucleic Acids Res. 2015 Oct 4;43(21):10546–59. doi: 10.1093/nar/gkv1005 (PMC4666351; doi:10.1093/nar/gkv1005)
Supplement: SUPPLEMENTARY DATA [file supp_gkv1005_nar-02513-h-2015-File015.pdf]

Table S1. Candidate AraR-binding sites in the genomes of *Bacteroides* and *Prevotella* spp.

Genome / Target gene	Site position	Site score	AraR locus tag / AraR site	Regulated operon	Other regulators
**Bacteroides thetaiotaomicron VPI-548**			**BT0354**		
BT0356	-83	5.64	AgAGTGTAttTGaTACACcaa	araMPRDAB-X-abf3	
	-59	5.85	AAAGTGTtACTtTTACACcca		
BT0365	-166	5.5	AAAGTGTAAaAaagACACTTa	X-susCD-susCD-abn1	HTCS-Ara-1
BT3047	-228	5.04	AtAaTGTAAaAtcTACACTca	X-susCD-X	HTCS-Ara-2
BT4163	-330	4.96	AAAtTGTAttAtcTACACTcT	X-susCD-XX-susCD-pelL-X	HTCS-Rgu-2
**Bacteroides ovatus ATCC 8483**			**BACOVA_01713**		
BACOVA_01715	-83	5.64	AgAGTGTAttTGaTACACcaa	araMPRDAB-X-abf3	
	-59	5.85	AAAGTGTtACTtTTACACcca		
BACOVA_03483	-139	5.09	AAAGTGTAtaTtTTACcCTTg	abf3-agaL	
**Bacteroides cellulosilyticus DSM 14838**			**BACCELL_05606**		
BACCELL_05604	-85	5.5	cAAGTGTtACTaaTACACTca	araMPRDAB-X-abf3	
	-61	5.85	AAAGTGTtACTtTTACACcca		
BACCELL_00858	-40	4.81	tAtGTGTAttTtTgACACcca	abn3-agaL-abf3-abf4-bgaL-abn4	
**Bacteroides cellulosilyticus WH2**			**BACWH2_0841**		
BACWH2_0839	-85	5.19	cAAGTGTAgtTaaTACACTca	araMPRDAB-X-abf3	
	-61	5.85	AAAGTGTtACTtTTACACcca		
BACWH2_0835	-358	4.98	AAAaTGTAttTtcTACACTTa	susCD-X-abn1	HTCS-Ara-1
BACWH2_1534	-37	4.81	tAtGTGTAttTtTgACACcca	abn3-agaL-abf3-abf4-bgaL-abn4	
**Bacteroides caccae ATCC 43185**			**BACCAC_02303**		
BACCAC_02305	-83	5.64	AgAGTGTAttTGaTACACcaa	araMPRDAB	
	-59	5.85	AAAGTGTtACTtTTACACcca		
**Bacteroides dorei DSM 17855**			**BACDOR_03632**		
BACDOR_03634	-121	5.38	AAAGTGTAggAaaTACACTTT	araMPRDAB	
	-58	5.79	AtAGTGTtACTtTTACACcca		
**Bacteroides eggerthii DSM 20697**			**BACEGG_01534**		
BACEGG_01536	-83	5.74	AAAGTGTAtaTaTTACACcaa	araMPRDAB-X-abf3	
	-59	5.58	AAAGTGTtACTtTTACACccg		
BACEGG_00165	-47	5.77	AAgGTGTtAtTtTTACACTcT	agaL	
**Bacteroides faecis MAJ27**			**KCYDRAFT_02805**		
KCYDRAFT_02807	-83	5.39	cAAGTGTAttTGaTACACcaa	araMPRDAB-X-abf3	
	-59	5.85	AAAGTGTtACTtTTACACcca		
KCYDRAFT_02813	-166	5.01	gAAGTGTAAaAaagACACTTa	X-susCD-susCD-abn1	HTCS-Ara-1
**Bacteroides finegoldii DSM 17565**			**BACFIN_07055**		
BACFIN_07057	-59	5.58	AAAGTGTtACTtTTACACccg	araMPRDAB-X-abf3	

**Bacteroides gallinarum DSM 18171**			**C233DRAFT_02383**		
C233DRAFT_02381	-85	5.84	AAAGTGTAtaTaTTACACTaa	araMPRDAB-X-abf3	
	-61	5.58	AAAGTGTtACTtTTACACccg		
C233DRAFT_02377	-358	5.01	AAAcTGTActTtTTACACTTa	susCD-X-abn1	HTCS-Ara-1
C233DRAFT_03386	-364	5.58	AAAGTGTtAtTtTTACACTcg	agaL	
**Bacteroides intestinalis DSM 17393**			**BACINT_02780**		
BACINT_02778	-85	5.29	cAAGTGTAgCTaaTACACTca	araMPRDAB-XX-abf3	
	-61	5.85	AAAGTGTtACTtTTACACcca		
BACINT_02771	-150	4.85	tAAtTGTAAtTtTcACACTTa	X-susCD-susCD-abn1	HTCS-Ara-1
BACINT_00345	-38	4.81	tAtGTGTAttTtTgACACcca	abn3-agaL-abf3-abf4-bgaL-abn4	
BACINT_03575	-116	4.75	tgAGTGTAttTcaaACAaTaa	abf5	
**Bacteroides oleiciplenus YIT 12058**			**HMPREF9447_03620**		
HMPREF9447_03618	-85	5.03	gtAGTGTAgCTaaTACACTca	araMPRDAB-X-abf3	
	-61	5.85	AAAGTGTtACTtTTACACcca		
HMPREF9447_03611	-153	4.74	tAAtTGTAgtTtcTACACTTa	X-susCD-susCD-abn1	HTCS-Ara-1
HMPREF9447_03041	-47	5.42	gAAGTGTtAtTtTTACACTTT	abn3-agaL-abf3-abf4-bgaL-abn4	
**Bacteroides plebeius DSM 17135**			**BACPLE_02213**		
BACPLE_02214	-44	5.83	tAAGTGTtACTtTTACACcaT	araMRDAB	
BACPLE_02216	-93	5.77	ttAGTGTtACTaaTACACTTT	araP	
BACPLE_03732	-60	4.8	tAgtTGTAtgTGaTACACcaa	abn3-abf3	
**Bacteroides salanitronis DSM 18170**			**Bacsa_0720**		
Bacsa_0722	-97	5.15	ttAGTGTAgaTagTACACTaa	araPMRDAB-abf3-abf2	
**Bacteroides salyersiae CL02T12C01**			**HMPREF1071_02469**		
HMPREF1071_02471	-83	5.84	AAAGTGTAAtAtTTACACcaa	araMPRDAB	
	-59	5.85	AAAGTGTtACTtTTACACcca		
**Bacteroides uniformis ATCC 8492**			**BACUNI_01359**		
BACUNI_03327	-97	4.92	tAAGTGTAtaTGTTACACaac	abn3-agaL-abf3-X-abf4-bgaL	
	-47	5.48	AtAGTGTtAaTtTTACACccg		
BACUNI_01357	-28	4.71	AAAGTGTAcagccTACACTaa	araMPRDAB	
	-4	5.85	AAAGTGTtACTtTTACACcca		
**Bacteroides vulgatus ATCC 8482**			**BVU_2322**		
BVU_2324	-121	5.38	AAAGTGTAggAaaTACACTTT	araMPRDAB	
	-58	5.77	AtAGTGTtACTtTTACACcaa		
**Bacteroides xylanisolvens XB1A**			**BXY_15620**		
BXY_15640	-83	5.64	AgAGTGTAttTGaTACACcaa	araMPRDAB-X-abf3	
	-59	5.85	AAAGTGTtACTtTTACACcca		
BXY_15680	-153	5.2	AAgcTGTAttTtTTACACTTa	susCD-X-abn1	HTCS-Ara-1

BXY_29680	-139	5.09	AAAGTGTAtaTtTTACcCTTg	abf3-agaL
**Prevotella albensis DSM 11370**			**K333DRAFT_01610**	
K333DRAFT_01608		6.32	AAaGTGTtAAtTTtACACcTT	araMPRDAB-abf3
**Prevotella paludivivens DSM 17968**			**B045DRAFT_01160**	
B045DRAFT_01162		6.5	AAAGTGTtAAtTTtACACTTT	araMPRDAB-abf3
**Prevotella bryantii C21a**			**G638DRAFT_00205**	
G638DRAFT_00203		5.85	aAAGTGTtaaaaagACACTTc	araMPRDAB-abf3
**Prevotella oris DSM 18711**			**D468DRAFT_01535**	
D468DRAFT_01533		5.82	aaAGTGTtaAtTagACACTca	araMPRDAB-abf3
**Prevotella copri CB7, DSM 18205**			**PREVCOP_04057**	
PREVCOP_04055		5.76	AtAGTGTtaaaaagACACTtT	araMPRDAB-abf3
**Prevotella saccharolytica JCM 17484**			**JCM17484DRAFT_02107**	
JCM17484DRAFT_02105		5.66	aAaGTGgtAAtTTtaCACcTa	araMPRDAB-abf3
**Prevotella ruminicola 23**			**PRU_0422**	
PRU_0424		5.65	AAGGTGTgtttttACACCTT	araMPRDAB-abf3
**Prevotella dentalis DSM 3688**			**HMPREF9136_1913**	
HMPREF9136_1911		5.61	AAAGtGTgaaaaatACcCTTT	araMPRDAB-abf3
**Prevotella maculosa DSM 19339**			**H164DRAFT_00687**	
H164DRAFT_00685		5.45	aAagTGTTaAtTaAACAtgTa	araMPRDAB-abf3
**Prevotella multisaccharivorax PPPA20, DSM 17128**			**Premu_2666**	
Premu_2668		5.08	aGaGTGTtaaaaatACACaCc	araMPRDAB-abf3

BACUNI_01357
